# Epigenetic profiling of circulating cell-free DNA for early detection and minimal residual disease assessment in lung cancer: a focus on DNA methylation

**DOI:** 10.3389/fonc.2026.1919279

**Published:** 2026-08-27

**Authors:** Tancredi Didier Bazan Russo, Valerio Gristina, Marco Donatello Delcuratolo, Leonardo Tamburi, Francesco Delli Muti, Filomena Mangiacotti, Clarissa Mujacic, Daniele Fanale, Antonio Galvano, Stefania Gori, Lorena Incorvaia, Antonio Giordano, Francesco Pepe, Umberto Malapelle, Antonio Russo, Lucia Anna Muscarella, Federico Pio Fabrizio

**Affiliations:** 1 Department of Precision Medicine in Medical, Surgical and Critical Care (Me.Pre.C.C.), Section of Medical Oncology, University of Palermo, Palermo, Italy; 2 Division of Medical Oncology, Department of Internal Medicine, The Ohio State University Comprehensive Cancer Center - Arthur G. James Cancer Hospital and Richard J. Solove Research Institute, Columbus, OH, United States; 3 Medical Oncology Unit, Fondazione Istituto di Ricovero e Cura a Carattere Scientifico (IRCCS) Casa Sollievo della Sofferenza, San Giovanni Rotondo, FG, Italy; 4 Catholic University of the Sacred Heart, Rome, Italy; 5 Lung Cancer Research Unit, Fondazione Istituto di Ricovero e Cura a Carattere Scientifico (IRCCS) Casa Sollievo della Sofferenza, San Giovanni Rotondo, FG, Italy; 6 Medical Oncology Unit, Istituto di Ricovero e Cura a Carattere Scientifico (IRCCS) Sacro Cuore Don Calabria Hospital, Negrar di Valpolicella, Verona, Italy; 7 Sbarro Institute for Cancer Research and Molecular Medicine and Center of Biotechnology, College of Science and Technology, Temple University, St. Philadelphia, PA, United States; 8 Department of Medical Biotechnology, University of Siena, Siena, Italy; 9 Department of Public Health, University Federico II of Naples, Naples, Italy; 10 Department of Medicine and Surgery, University of Enna “Kore”, Enna, Italy

**Keywords:** cell-free DNA, DNA methylation, early detection, epigenetics, liquid biopsy, lung cancer, machine learning, minimal residual disease

## Abstract

Lung Cancer (LC) continues to be the biggest cause of cancer-related deaths around the world, mostly because of delayed diagnosis. Even if tissue biopsies and circulating tumor DNA (ctDNA) tests have revolutionized clinical management of LC patients, their effectiveness is restricted in settings with lower tumor burden, molecular heterogeneity, and bias in sampling approaches. In this scenario, the epigenetic profiling of cell-free DNA (cfDNA) stands out as a promising, less invasive approach, accurately detect cancer traces. Evidence from stage I-II disease and CT-detected pulmonary nodules supports the diagnostic potential of cfDNA methylation, although further validation in prospective screening cohorts remains necessary. Beyond genomic alterations, cfDNA epigenetic changes, including DNA methylation, chromatin organization, nucleosome positioning, and fragmentation patterns, reflect multi-dimensional complexity of tumor biology. These properties convey both the functional status and the origin of the circulating DNA fragments, accelerating for tumor integrating genomic analysis. Within this group, DNA methylation is the biologically robust and clinically well-established epigenetic marker, as alterations in methylation linked to cancer often occur in the early stages of tumorigenesis and are commonly found across different cancer cell types. Here, we explored the biological and clinical relevance of the epigenetic landscape of cfDNA in LC patients, particularly focusing on DNA methylation-based biomarkers and their evolving applications towards early diagnosis and post-surgical monitoring of minimal residual disease (MRD). We aimed to comprehensively overview analytical approaches for cfDNA methylation analysis, including targeted and genome-wide profiling strategies, and discuss their integration with machine learning (ML) and multi-omics frameworks in order to improve diagnostic performance and clinical applicability in LC management.

## Graphical Abstract

**Figure:**
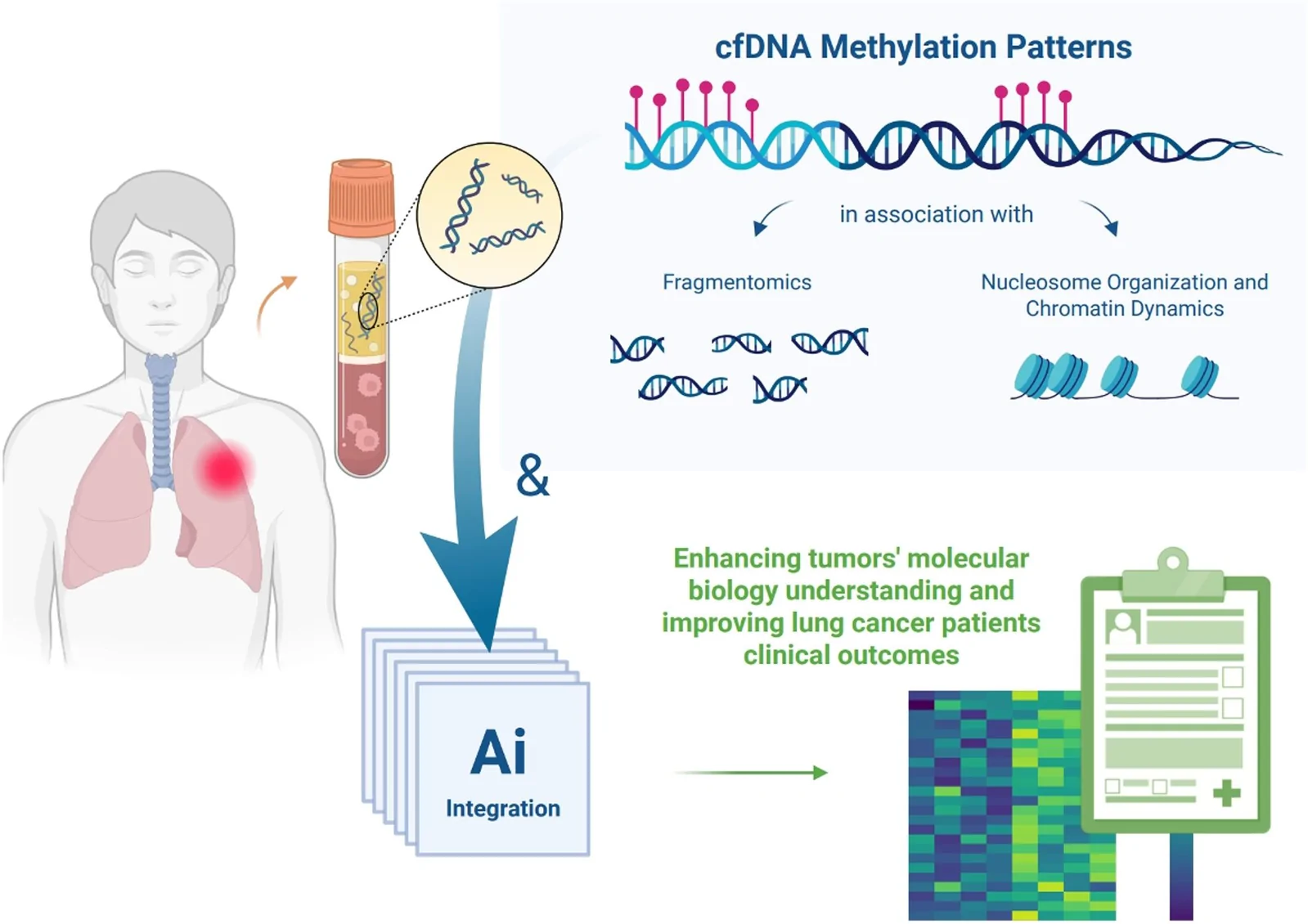

## 1 Introduction

Lung cancer (LC) remains one of the most commonly diagnosed malignancies worldwide and the leading cause of cancer-related deaths (1). Non-Small Cell Lung Cancer (NSCLC) accounts for 85% of cases, and more than 75% of patients are diagnosed with advanced-stage disease (2). Tissue biopsy has been elected as gold standard for the diagnosis and molecular profiling of LC; however, it has several limitations depending on invasiveness, molecular heterogeneity and limitations in “scant” sample processing and molecular profiling.

The advent of liquid biopsy (LB) has significantly changed the management of LC patients, when tissue samples are unavailable or inadequate, enabling longitudinal monitoring of tumor evolution and real-time monitoring residual traces. LB encompasses several circulating tumor-derived analytes, such as cell-free DNA (cfDNA), circulating tumor DNA (ctDNA), Circulating Tumor Cells (CTCs), Tumor-Educated Platelets (TEPs) and Extracellular Vesicles (EVs) (3). In the early-stage setting, low-dose computed tomography (LDCT) screening has been shown to reduce LC mortality approximately 20%; however, false-positive rate of 96.4%, which may compromise clinical sustainability. In addition, cumulative radiation exposure and invasive procedures are also limitations (4, 5). Several studies have demonstrated that non-invasive biomarkers could improve diagnostic performance, sensitivity and specificity, over LDCT alone (6, 7).

Furthermore, the rapid change of therapeutic scenario with the introduction of targeted therapy and immunotherapy into earlier stages of disease has significantly improved clinical outcomes in perioperative cases. However, a significant proportion of patients still experience relapse, underscoring the need to detect “traces” of tumor to early identify recurrent patients before imaging techniques (8). Several studies across the neoadjuvant, perioperative and adjuvant context demonstrated the prognostic and predictive role of ctDNA clearance, supporting the clinical implementation in early-stage disease (9, 10). Moreover, ctDNA analysis in early-stage disease remains challenging due to limited and heterogeneous shedding of tumor-derived DNA in the bloodstream, producing low variant allele fractions and relatively high false-negative rate. To overcome these limitations, epigenetic biomarkers are becoming increasingly as complementary tools (11).

Aberrant DNA methylation contributes to the silencing and the dysregulation of tumor suppressor genes and oncogenes, respectively. Epigenetic biomarkers offer many advantages, even when detected via LB. First, DNA methylation occurs at an early phase of tumorigenesis, sometimes preceding detectable genetic mutations (11, 12). Second, it is a tissue-specific mechanism; indeed, methylation levels in tumor cells are different from normal cells and significantly vary between different histological types (11, 13, 14). Third, DNA methylation is biologically stable and can be reliably quantified using methylation-specific PCR techniques (11). Finally, whereas mutation-based approaches interrogate a limited number of genomic loci, methylome analyses assess between thousands to millions of cytosine-phosphate-guanine (CpG) sites across the whole genome. Contiguous CpG sites exhibit synchronized methylation patterns, allowing tumor signals to be detected even when only a subset of loci is analyzed, thus potentially improving analytical sensitivity compared to mutation-based approaches that require detection of individual variants (15). This difference is particularly significant in early-stage cancers, where the fraction of ctDNA can be extremely low and the likelihood of detecting individual somatic mutations may be limited by stochastic sampling effects. In contrast, DNA methylation alterations often manifest as coordinated regional changes involving multiple CpG sites and extensive genomic regions, generating broader and redundant molecular signals that can be detected even from small amounts of tumor-derived cfDNA. Therefore, even when only a small fraction of tumor-derived cfDNA is present in circulation, the likelihood of detecting a tumor-specific methylation pattern may remain higher than that of identifying a single low-frequency somatic mutation (7, 16, 17). This advantage is supported by the biological nature of cancer-associated DNA methylation alterations, which often arise in the early stages of tumorigenesis and involve large genomic regions, providing a more comprehensive picture of the molecular changes associated with the tumor than isolated somatic mutations (18, 19).

Consequently, cfDNA methylomics often yields a higher signal-to-noise ratio than purely genomic approaches, thereby improving sensitivity in the early diagnosis and in the detection of MRD. In LC, DNA methylation biomarkers such as *prostaglandin E receptor 4 (PTGER4), Ras-binding domain-containing protein 1A (RASSF1A), death-associated protein kinase (DAPK), P16* and *short stature-associated homobox 2 (SHOX2)* can be detected in the early-stages with high sensitivity and specificity (11).

We sought to provide a comprehensive overview of the current evidence on the epigenetic profiling of circulating cfDNA for early detection of LC, with an important focus on its emerging role on MRD assessment. Particularly, we discuss the biological basis of epigenetic alterations in cfDNA, the main technological platforms used for their detection, and the available clinical evidence supporting the use of methylation-based biomarkers for LC screening, early diagnosis and MRD monitoring. In addition, we summarize the current challenges and future perspectives for integrating epigenetic LB approaches into routine clinical practice.

## 2 cfDNA methylation biomarkers in LC: biological basis, analytical considerations, and clinical evidence

cfDNA epigenetics represents a compelling area of research, spanning from DNA methylation patterns to histone modifications and structural organization of chromatin signals, which shape downstream transcriptional and post-translational regulatory processes (20–22).

It is important that epigenetic signals, including methylation, fragmentation, and nucleosome-related signals, can trace the tissue of origin, map tumor private epigenomic signatures, and perform cellular deconvolution of cfDNA, as well as the profiling of biological and cellular heterogeneity (23). The biological basis of these epigenetic signatures stems from the profound remodeling of the tumor epigenome that occurs during malignant transformation. Early tumor development is accompanied by coordinated alterations in DNA methylation, including focal hypermethylation of promoters, global hypomethylation, and disruption of lineage-specific methylation programs, which contribute to transcriptional reprogramming, cellular plasticity, and tumor evolution. Unlike isolated genetic alterations, epigenetic changes can affect large genomic regions and thus generate distributed molecular patterns that reflect the biological identity and functional state of cancer cells (18, 19). As a result of tumor cell turnover, cfDNA is released into the circulation primarily through apoptosis and necrosis, although active secretion mechanisms may also contribute. During this process, circulating DNA fragments retain relevant characteristics of the original chromatin environment, including methylation profiles, nucleosome organization, and fragmentation patterns. Therefore, cfDNA represents not only a quantitative source of tumor-derived material but also a molecular archive of tumor-associated epigenomic states, providing the biological rationale for its application in cancer diagnosis and disease monitoring (24, 25).

Interest in cfDNA has grown due to its ability to retain epigenetic and structural hallmarks of its cell of origin, such as DNA methylation profiles, chromatin accessibility, nucleosome occupancy, and fragmentation patterns. Collectively, these features are referred to as “epigenetic footprints”, which are indicative of the transcriptional and chromatin landscapes of the originating tissue and serve as biologically informative signatures for cancer detection (26, 27). These biological properties make approaches based on cfDNA methylation particularly promising in clinical settings characterized by a low tumor burden, such as early diagnosis and monitoring of minimal residual disease, although their effectiveness continues to depend on the tumor fraction, test sensitivity, and biomarker selection (28, 29).

A comprehensive characterization of cfDNA methylomes, non-random fragmentation patterns, and nucleosome-associated signals can therefore provide complementary information on tissue identity, chromatin organization, and tumor-specific biological states (30). To enhance the translational relevance of cfDNA methylation profiling in LC, **Table 1** summarizes representative methylation-based biomarkers that have been the subject of clinical studies, highlighting their biological rationale, reported diagnostic performance, current validation status, and potential clinical applications. Although several methylation biomarkers have demonstrated promising analytical performance, prospective multicenter validation, test standardization, and evaluation in real-world clinical settings remain essential before they can be routinely implemented.

**Table 1:** Representative biomarkers of cfDNA methylation in LC: biological basis, clinical validation status, and potential applications.

Biomarker / methylation signature	Biological rationale	Analytical approach	Reported diagnostic performance	Clinical validation status	Potential clinical applications
***SHOX2*** ***(***31–33)	Hypermethylation of the *SHOX2* gene at the promoter level is a common epigenetic alteration LC and reflects abnormal regulation of differentiation and tumor-associated transcriptional programs	MSP, qMSP, targeted cfDNA methylation assays	The reported sensitivity ranges from approximately 60% to 80%, with a specificity that often exceeds 90%, although performance varies depending on the type of sample, the stage of the disease, and the test method; diagnostic accuracy was found to be improved when combined with additional methylation markers	One of the most widely studied LC methylation biomarkers; validated in independent tissue and plasma/cfDNA cohorts	LC diagnosis, differential diagnosis of pulmonary nodules, complementary biomarker for imaging-based screening
***RASSF1A*** (31–33)	Hypermethylation of *RASSF1A* at promoter region leads to the epigenetic silencing of a tumor TSG involved in cell cycle regulation, apoptosis, and genomic stability	MSP, qMSP, bisulfite-based methylation assays, plasma cfDNA analysis	Sensitivity generally ranges from 40% to 70%, while specificity is often greater than 85%, depending on the cohort and the test used	One of the earliest and most extensively studied methylation biomarkers for LC; limited sensitivity as a single marker due to tumor heterogeneity	Diagnostic panels, molecular classification, prognostic assessment
***PTGER4*** (32, 34)	Abnormal promoter methylation of *PTGER4* contributes to transcriptional dysregulation during lung carcinogenesis and reflects tumor-specific epigenetic remodeling	qMSP, targeted methylation assays, cfDNA methylation profiling	The reported diagnostic performance has been promising, with AUC values generally ranging from approximately 0.75 to 0.90 in selected cohorts	Investigated in tissue and circulating DNA studies, however, independent prospective validation remains limited	Early diagnosis, integration into multi-marker methylation panels, risk stratification
***DAPK*** ***(***35, 36)	Hypermethylation of *DAPK* promotes the epigenetic silencing of a tumor suppressor involved in apoptosis, cytoskeletal regulation, and cellular death pathways, and is frequently observed during lung carcinogenesis	MSP, qMSP, bisulfite-based methylation assays, plasma/cfDNA methylation analysis	Diagnostic performance varies from one study to another: sensitivity is generally limited when the marker is evaluated on its own, while high specificity is often reported; performance improves when combined with other methylation biomarkers	One of the first methylation markers for LC to be studied; its biological relevance has been established, but its clinical application as a standalone biomarker remains limited	Multimarker methylation panel component for early diagnosis, molecular characterization, and risk stratification
***CDKN2A /p16*** ***(***37, 38)	Hypermethylation of the *CDKN2A/p16* gene at promoter region leads to the transcriptional silencing of an important TSG that regulates the G1–S cell cycle checkpoint and contributes to uncontrolled cell proliferation in LC	MSP, qMSP, methylation-specific assays, targeted bisulfite sequencing	The reported sensitivity varies (approximately 30–60% depending on the cohort and the test), with relatively high specificity; diagnostic accuracy is improved when combined with additional markers	Extensive biological and clinical research has been conducted on NSCLC; however, the heterogeneity in methylation frequency limits its use as a single diagnostic biomarker	Early detection panels, prognostic evaluation, and molecular characterization of NSCLC
***APC*** ***(***31, 39, 40)	Hypermethylation of the *APC* promoter region leads to the epigenetic silencing of a regulator of the Wnt signaling pathway, contributing to malignant transformation	MSP, qMSP, targeted methylation assays	Variable diagnostic performance. Reported in subgroups of NSCLC cohorts, often as part of multi-marker methylation signatures rather than as an independent diagnostic biomarker	Solid biological evidence; clinical application is still in the experimental stage	Component of combined methylation diagnostic signatures
***SOX17*** ***(***31, 41)	Epigenetic silencing of *SOX17* due to promoter hypermethylation affects the developmental pathways and cell differentiation programs involved in tumor progression	qMSP, methylation arrays, targeted sequencing approaches	Moderate sensitivity as a single biomarker; improved performance when included in methylation panels	An experimental biomarker with a solid biological basis but limited prospective validation	Early detection and molecular characterization
***HOXA9*** ***(***31, 42)	Abnormal methylation of *HOXA9* is involved in development contributes to lung carcinogenesis and alterations in cellular identity	Targeted methylation assays, sequencing-based approaches	Several studies have linked *HOXA9* gene methylation to the diagnosis of NSCLC and to prognostic stratification	It requires further validation and analytical standardization in larger prospective cohorts	Prognostic assessment and multi-marker biomarker panels
***SEPT9*** ***(***43, 44)	Abnormal methylation of the *SEPT9* gene is a tumor-associated epigenetic alteration detectable in circulating DNA	Plasma methylation assays, qMSP, targeted methylation platforms	High diagnostic accuracy has been demonstrated primarily in the case of colorectal cancer; studies on LC show sensitivity and specificity that vary depending on the context	Clinically validated in other solid tumors; lung SqCC application remains investigational	Strategies for detecting multiple tumors and methylation-based screening approaches
**Multi-CpG methylation signatures / methylation panels** (16, 45, 46)	The integration of multiple tumor-associated CpG alterations enables the detection of widespread and coordinated epigenomic changes, increasing sensitivity compared to single-marker approaches and reducing the impact of tumor heterogeneity	WGBS, EPIC methylation arrays, cfMeDIP-seq, targeted methylation sequencing panels	Several studies report AUC values generally above 0.85–0.90, with better performance in the early stages of the disease and in settings characterized by a low tumor fraction, depending on the cohort and platform	There is growing clinical interest, supported by validation studies conducted on large retrospective cohorts; however, prospective multicenter validation and standardization are still needed	Early diagnosis, screening support, monitoring of MRD, surveillance for relapse, and prediction of the tissue of origin

APC, Adenomatous Polyposis Coli; AUC, Area Under the Curve; CDKN2A/p16, Cyclin-Dependent Kinase Inhibitor 2A; cfDNA, circulating cell-free DNA; cfMeDIP-seq, cell-free DNA methylated DNA immunoprecipitation sequencing; CRC, colorectal cancer; DAPK, Death-Associated Protein Kinase; EPIC, Infinium MethylationEPIC BeadChip; G1–S checkpoint, Gap 1–Synthesis cell cycle checkpoint; HOXA9, Homeobox A9; LC, lung cancer; MRD, minimal residual disease; MSP, methylation-specific polymerase chain reaction; NSCLC, non-small cell lung cancer; qMSP, quantitative methylation-specific polymerase chain reaction; RASSF1A, Ras Association Domain Family Member 1 Isoform A; SEPT9, Septin 9; SHOX2, Short Stature Homeobox 2; SOX17, SRY-Box Transcription Factor 17; TSG, tumor suppressor gene; WGBS, whole-genome bisulfite sequencing. The biomarker/methylation signature names indicated in bold are listed and defined below the table.

### 2.1 DNA methylation and cancer methylome

DNA methylation arises from the addition of methyl group (CH_3_) catalyzed by DNA methyltransferases (DNMTs) enzymes to the 5th carbon of cytosine residues, resulting in 5-methylcytosine (5mC) (47).

This epigenetic modification has attracted attention due to its central role in the regulation of gene expression, chromatin organization and cellular homeostasis. Aberrant DNA methylation patterns have been extensively documented across multiple solid tumors, including LC (21, 48). However, altered methylation profiles within CpG dinucleotides at promoter regions, can contribute into dysregulation at cell-cycle, differentiation and DNA repair mechanisms, thereby promoting lung tumor initiation, progression and therapy resistance (49).

CpG Island Methylator Phenotype (CIMP) has emerged as an important molecular feature for distinguishing tumoral subgroups characterized by aberrant promoter methylation. CIMP may be useful to add relevant insights into the complexity of tumor heterogeneity, including differences in immune-related transcriptional programs and therapeutic sensitivity (50).

Aberrant methylation of multiple genes commonly involved in the development of LC, as well as *RASSF1A, Adenomatous Polyposis Coli (APC), SHOX2, SRY-Box Transcription Factor 17 (SOX17)*, and *Homeobox A9 (HOXA9)*, has been associated with the deregulation of pathways implicated in cell growth, differentiation, and tumor suppression (31). Importantly, genome-wide methylation patterns can retain lineage-specific features, supporting that tumor cfDNA preserves epigenetic signatures.

Beyond the 5mC, the 5-hydroxymethylcytosine (5hmC) arises from another important epigenetic modification mechanism that contribute to cancer-specific epigenomic signatures and is the result of the oxidization of 5mC, during active DNA demethylation (51). The 5hmC is getting more attention as a standalone epigenetic marker for dynamic chromatin modification. Cancer cells tend to show a significant reduction and redistribution of 5hmC across the genome, in which may contribute to the alteration in the accessibility of chromatin and transcriptional regulation. In LC, tissue-specific hydroxymethylation signatures have been associated with epigenetic reprogramming depending on cells origin and tumor progression (52).

By shaping chromatin structure, transcription landscape, and cellular plasticity, epigenetic alterations could result into the development and persistence of malignant phenotypes, representing dynamic mechanism underlying LC development and progression (53).

### 2.2 cfDNA fragmentation patterns

Fragmentomics refers to the analysis of non-randomic fragmentation patterns of cfDNA resulting from apoptosis and necrosis (54). These circulating cfDNA fragments exhibit characteristic cleavage patterns, size distributions and sequence-dependent end motifs. At the genomic level, cfDNA fragments tend to be clustered at genome-specific locations, and their upstream and downstream sequence patterns might reflect the accessibility and high-order chromatin organization (55–57).

Beyond methylation profiling, fragmentomic patterns can provide complementary information for the epigenetic analysis of cfDNA. Tumor-derived cfDNA fragments can display distinctive fragmentation profiles, as well as reduced length, and altered flanking-end sequences compared to normal tissues (27). Specific fragmentation patterns reflect genome’s architecture and chromatin structure, which can be used to decode biologically relevant signals about the epigenetic state of tumor cells.

In this regard, cfDNA fragmentation may capture tumor-associated chromatin features and contribute to the characterization of biological diversity among cancer subtypes, regardless of the abundance of ctDNA (20, 57).

### 2.3 Nucleosome organization and chromatin dynamics

The positioning of nucleosomes, the structural architecture of chromatin, and histone modification patterns have been investigated as complementary epigenetic features in cfDNA analysis. Accordingly, cfDNA epigenetic analysis is expanding beyond DNA sequencing and methylation analysis to link circulating fragments to higher-order chromatin organization and histone-associated regulation (58). Although cfDNA fragments derived from the bloodstream, the properties of the chromatin’s native structure are preserved, as the histone-DNA complexes protect regions associated with nucleosomes. Consequently, cfDNA is particularly enriched in nucleosome-protected fragments, which contributes to the preservation of fragment integrity and influences cfDNA fragmentation patterns (59).

In this setting, transcription is closely intertwined with the nucleosome organization and chromatin accessibility (60). It has been reported that nucleosomal profiles may represent promising diagnostic biomarkers for tumor prediction and disease classification of NSCLC, with cfDNA length patterns reflecting underlying transcriptional activity and chromatin states (61). However, the biological and clinical applicability of these signatures require further investigation.

Furthermore, DNA methylation within CpG islands transcriptionally repressed may be accompanied by aberrant nucleosome positioning and increased chromatin condensation, thereby establishing a mechanistic link between epigenetic silencing and nucleosome occupancy (62).

At the same time, the integration of ML algorithms with cfDNA methylation patterns, fragmentomic and nucleosome-level chromatin features, computational deconvolution approaches, and multi-omics models, has expanded the potential of epigenomic molecular characterization. These computational strategies have shown substantial progress in improving early diagnosis, supporting MRD evaluation and monitoring, as well as the inference of cancer tissue of origin, which may be important for the clinical development LB-based cancer-detection tools (63).

## 3 Analytical approaches for epigenetic profiling

**Figure 1:**
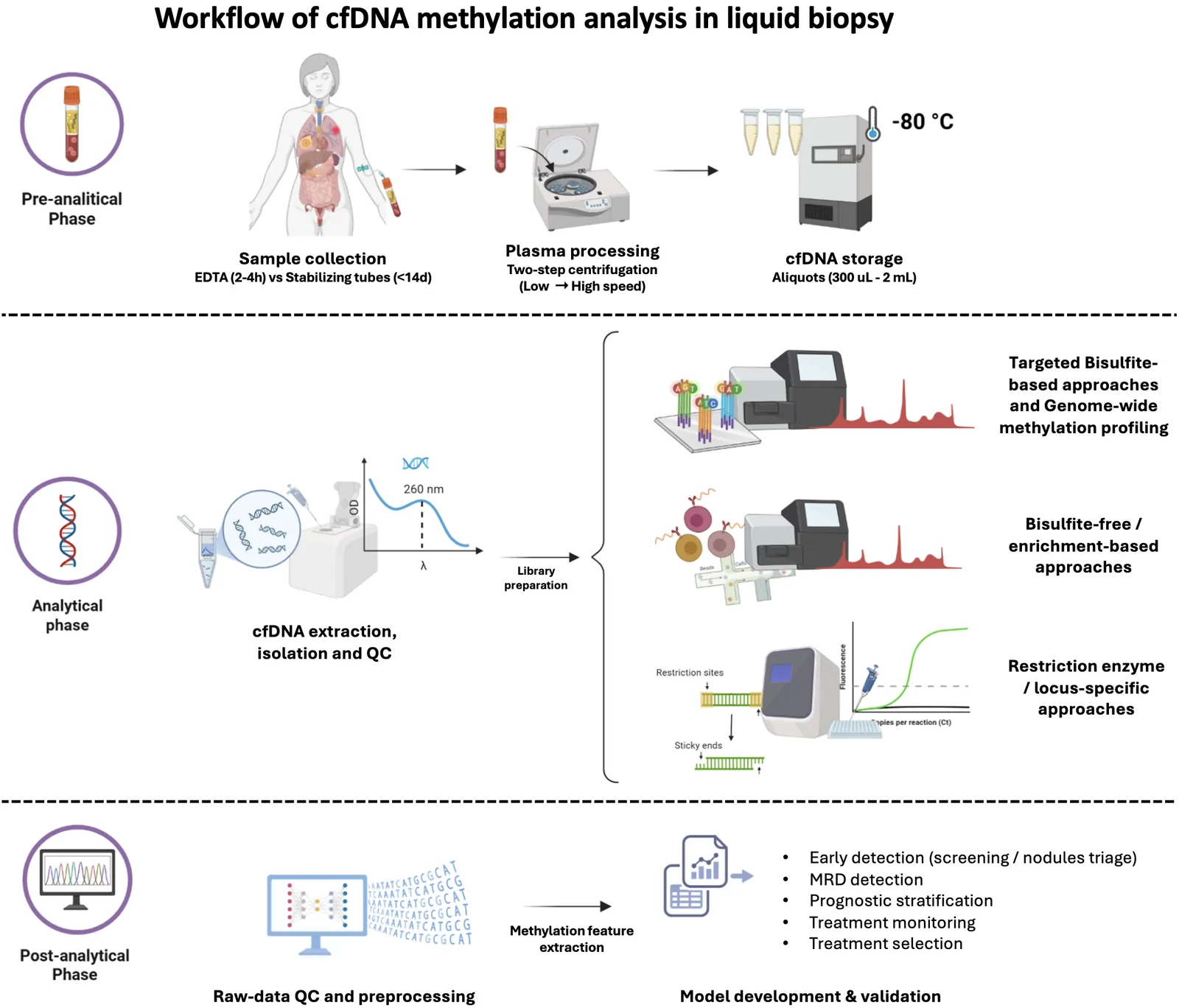
Workflow of cfDNA methylation analysis in LB. The workflow includes pre-analytical, analytical, and post-analytical phases. In the pre-analytical phase, blood is collected in EDTA or stabilizing tubes, followed by plasma separation and storage at −80 °C. In the analytical phase, cfDNA extraction, isolation and QC are followed by library preparation and methylation profiling using different approaches, including bisulfite-based methods (WGBS, RRBS and cfRRBS, MCTA-seq, qMSP, ddMSP, pyrosequencing, Methyl-Seq and cfMethyl-Seq, HM450K BeadChip and MethylationEPIC 850K, Heatrich-BS, BSPP), bisulfite-free enrichment approaches (cfMeDIP-seq, 5hmC-Seal), and restriction enzyme-based methods (MRE-seq, RECAP-seq, HELP). In the post-analytical phase, bioinformatic analysis and data processing generate clinically relevant outputs, including early detection, MRD assessment, prognostic stratification, treatment monitoring, and therapy selection. 5hmC, 5-hydroxymethylcytosine; cfDNA, cell-free DNA; cfRRBS, cell-free reduced representation bisulfite sequencing; cfMeDIP-seq, cell-free methylated DNA immunoprecipitation sequencing; cfMethyl-Seq, cell-free methylation sequencing; ddMSP, droplet digital methylation-specific PCR; EDTA, ethylenediaminetetraacetic acid; HELP, HpaII tiny fragment enrichment by ligation-mediated PCR; HM450K, Illumina HumanMethylation450 BeadChip; EPIC 850K, Illumina Infinium MethylationEPIC BeadChip; MCTA-seq, methylated CpG tandem amplification sequencing; MRE-seq, methylation-sensitive restriction enzyme sequencing; MRD, minimal residual disease; Methyl-Seq, methylation sequencing; BSPP, bisulfite padlock probe sequencing; NGS, next-generation sequencing; QC, quality control; qMSP, quantitative methylation-specific PCR; RECAP, Restriction Enzyme-based CpG-methylated fragment AmPlification; RRBS, reduced representation bisulfite sequencing; WGBS, whole-genome bisulfite sequencing.

cfDNA methylation profiling techniques can be broadly classified into the three main groups according to experimental procedures, as shown in **Figure 1**. The first approach concerns the bisulfite conversion; the second involves methods based on methylated or modified DNA without chemical conversion; and the third includes methylation-sensitive restriction endonucleases procedures. Several analytical platforms have been developed differing in terms of genomic coverage, analytical sensitivity, and their ability to process short and highly fragmented cfDNA molecules (64, 65).

The bisulfite-based assays rely on chemical process in which unmethylated cytosines are converted into uracil after sodium bisulfite treatment, whereas methylated cytosines (5mC) remain unaltered (66).

Whole-genome bisulfite sequencing (WGBS) enables comprehensive methylome profiling at single-nucleotide resolution, even from small DNA amounts (~125–250 pg). However, its clinical implementation remains challenging due to high sequencing cost and variable DNA recovery efficiency (67).

Reduced representation bisulfite sequencing (RRBS) and its cfDNA-adapted form (cfRRBS) have been developed to overcome some of these limitations. By targeting CpG-rich regions, these approaches reduce sequencing requirements while preserving single-base resolution. Unfortunately, these approaches require comparatively higher-quality DNA input (≥10 ng) and provide only partial coverage of the global methylome (68). At the ultra-low-input end of the range, methylated CpG tandem amplification and sequencing (MCTA-seq) specifically targets and amplifies CGGCGG-rich CpG regions from very small amount of DNA (~7.5 pg), achieving high analytical sensitivity. Nevertheless, its restricted genomic depth and potential sequence-dependent bias represent important limitations (69).

Specific locus-based bisulfite tests, as well as quantitative methylation-specific PCR (qMSP) and digital droplet methylation-specific PCR (ddMSP), have been mostly investigated for clinical translation. QMSP enables quantitative detection of differential methylated regions (DMRs) using low DNA amounts (~20–100 ng). However, its poor multiplexing capability may restrict broader clinical applications (65).

Compared with qMSP, ddMSP provides increased analytical sensitivity by favoring the detection of rare methylation changes from low-input cfDNA samples (10–50 ng). However, its implementation may depend by assay complexity and the requirement for highly optimized locus-specific designs (63). Pyrosequencing acts on single-stranded DNA, whereas ddMSP specifically targets double-stranded DNA; however, both methods offer enhanced sensitivity, rendering them particularly well-suited for use with low-input samples and resulting in cost-effective outcomes. Although pyrosequencing is widely used, its limited sensitivity (typically detecting methylated alleles above 5%) constrains its application in samples with very low-abundance tumors (64).

Targeted bisulfite sequencing methods provide resolution-rich methylation patterns at predefined loci, generally require approximately 50 ng of input DNA. Their distinctiveness mainly depends on an optimized primer and probe design, enabling accurate quantification of selected molecular targets (70).

Likewise, Methyl-Seq and cfMethyl-Seq workflows are tailored for CpG-rich cfDNA fragments and incorporate unique molecular identifiers (UMIs) to boost quantitative reliability requiring ~5–10 ng of DNA input. In fact, these approaches are affected by DNA degradation and potential sequence bias introduced during bisulfite-based conversion (71, 72).

Arrays-based platforms, including the HM450K BeadChip and MethylationEPIC (HM850K), can enable specific CpG dinucleotides with high throughput analysis using small input amounts of DNA (~10 ng). However, their restricted genomic coverage may hinder their ability to identify novel methylation biomarkers and perform discovery-oriented applications (73). Additional specialized bisulfite-based procedures encompass Heatrich-BS, which selectively enriches CpG-rich cfDNA fragments through very small input quantity (≈5–10 ng), although with limited genomic coverage (74). Conversely, bisulfite padlock probe sequencing (BSPP) achieves high-specificity through probe-based targeting using approximately 10–50 ng of input DNA but requires extensive locus-specific assay optimization (75).

Bisulfite-free enrichment methods bypass chemical modification and instead isolate methylated or specifically modified DNA fragments through affinity-based capture approaches, preserving cfDNA integrity and reducing DNA degradation related to the conversion (76). Among these, cell-free methylated DNA immunoprecipitation-sequencing (cfMeDIP-seq) relies on antibodies targeting 5-methylcytosine to capture methylated DNA fragments (up to 10 ng) (77). However, these approaches provide lower genome resolution and may be susceptible to enrichments-related bias.

The 5hmC-Seal technique specifically profiles 5-hydroxymethylcytosine (5hmC), enabling high-sensitivity detection from ultra-traceable amounts of cfDNA (~1–5 ng) (78, 79),.

Although these enrichment-based methods are undoubtedly low-input and preserve cfDNA fragment integrity by avoiding bisulfite conversion, they do not achieve single-base pair resolution and may be affected by capture efficiency variability and enrichment-related bias (80). While these capture methods have proven to be effective in exploring specific genomic and epigenomic hallmarks, a wider and more comprehensive characterization of the whole genome, including cfDNA-associated alterations, is still needed.

Among alternative methylation profiling strategies, restriction enzyme-based sequencing should be mentioned. These methods rely on methylation-sensitive restriction enzymes that specifically cut DNA, resulting in a thorough genome-wide analysis. Methylation-sensitive restriction enzyme sequencing (MRE-seq) captures unmethylated CpG regions without requiring bisulfite conversion; however, its application may be limited by reduced genomic coverage and the challenging interpretation of highly fragmented DNA (ranging from 10 to 50 ng of DNA input) (81),.

An emerging approach, Restriction Enzyme-based CpG-methylated fragment AmPlification sequencing (RECAP-seq), combines enzymatic methylation sequencing (EM-seq)-based library preparation with digestion by using restriction enzyme BstUI, which selectively recognizes CGCG motifs enriched in CpG islands. This strategy enables selective enrichment and amplification of hypermethylated DNA fragments and may accurately improve the identification of cancer-associated epigenetic markers from the circulating DNA (82). In addition to this, HELP (HpaII tiny fragment Enrichment by Ligation-mediated PCR) assay relies on restriction enzyme-mediated digestion followed by locus-specific analysis of DNA methylation patterns. Nevertheless, these assays exhibit a reduced bias with respect to CpG islands and offer a more comprehensive coverage of genomic regions with lower CpG density (83).

Overall, cfDNA methylation profiling encompasses a spectrum of approaches ranging from genome-wide discovery tools to highly targeted assays designed for clinical applications (63). Bisulfite-based methods are the most accurate for high-resolution methylation profiling and cover broad genomic regions. Conversely, enrichment-based methods are more effective at preserving cfDNA and suitable for low-input samples. Restriction enzyme-based approaches occupy an intermediate position, offering relatively rapid and straightforward workflows for the detection of predefined methylation patterns biomarkers, even though their genomic coverage remains limited (65, 84).

Taken together, these complementary approaches provide valuable tools for achieving timely and more functional early cancer diagnosis, risk stratification, prognostic assessment, and clinical translation of cfDNA-based epigenetic biomarkers.

### 3.1 Comparative analysis of main platforms for cfDNA methylation profiling: analytical performance, limitations, and clinical application

Although several technologies have been developed for cfDNA methylation profiling, their analytical performance and clinical applicability vary substantially depending on the intended clinical purpose. Genome-wide approaches, including WGBS, offer the highest analytical resolution, enabling unbiased identification of tumor-specific methylation profiles, epigenetic subtypes, and tissue-of-origin signatures at single-CpG resolution (85). Bisulfite sequencing remains the gold standard at the genomic level for DNA methylation profiling, thanks to its ability to provide single-CpG resolution and comprehensive coverage of the methylome; however, conventional bisulfite conversion can cause DNA degradation, which represents a potential limitation in the analysis of highly fragmented cfDNA.

Compared to targeted approaches, WGBS offers superior genomic coverage and discovery capabilities; however, its higher sequencing requirements, greater costs, complex bioinformatics processing, and reduced feasibility in samples with extremely low tumor fractions pose significant barriers to clinical translation (67, 86). Therefore, these approaches are primarily used for biomarker discovery, molecular classification, and biological characterization rather than for routine diagnostic applications (87). Alternative genomic-level strategies, such as EM-seq, have been developed to overcome certain limitations of bisulfite-based methods, using enzymatic conversion approaches that preserve DNA integrity while maintaining high accuracy in methylation detection. Compared to conventional bisulfite sequencing, EM-seq may offer better performance for fragmented cfDNA; however, its clinical implementation remains limited by sequencing requirements, technical complexity, and the need for further analytical and clinical validation. In contrast, targeted methylation strategies, including qMSP, ddMSP, and targeted bisulfite sequencing panels, represent more clinically viable alternatives, as they offer a more favorable balance between analytical sensitivity, DNA input requirements, turnaround time, and compatibility with standard molecular diagnostics laboratories compared to genome-wide approaches (65, 88). Compared to genome-wide bisulfite sequencing and EM-seq, targeted methylation panels offer reduced genomic coverage but greater scalability, lower costs, faster turnaround times, and greater suitability for routine clinical workflows. For example, methylation tests targeting loci associated with LC, such as *SHOX2*, *RASSF1A*, *PTGER4*, *DAPK*, and *CDKN2A/p16*, have demonstrated promising diagnostic performance for the detection of LC and its molecular characterization (11, 31, 89). However, single-marker tests are less robust than multi-marker approaches, as their performance can be affected by biological heterogeneity, differences in methylation patterns among histological subtypes, and variable loss of tumor DNA.

Compared to single-marker tests, targeted multi-CpG panels offer greater analytical robustness by incorporating multiple tumor-associated regions, thereby increasing sensitivity and reducing the impact of tumor heterogeneity, particularly in samples with low tumor burden (31, 45). Therefore, while genome-wide approaches maximize the potential for discovery, targeted multi-CpG panels currently represent the most advantageous balance between analytical performance and clinical feasibility for diagnostic applications. Bisulfite-free enrichment methods, including cfMeDIP-seq and 5hmC-Seal, offer significant advantages for highly fragmented cfDNA, as they prevent bisulfite-induced DNA degradation and preserve the integrity of the fragments. Compared to bisulfite-based methods, these approaches may offer greater compatibility with highly fragmented cfDNA; however, they generally compromise single-CpG resolution and require further standardization of enrichment efficiency, antibody specificity, CpG density, and normalization strategies. These approaches have demonstrated their utility for cancer detection, inference of tissue of origin, and biological classification; however, their analytical interpretation is influenced by enrichment efficiency, antibody specificity, CpG site density, and normalization strategies. Furthermore, the lack of single-base resolution may limit their application when precise quantification of methylation at specific loci is required (80, 90). In particular, 5hmC-based approaches have provided additional biological insights by revealing dynamic epigenomic states associated with cellular identity and tumor-associated transcriptional programs (91).

Similarly, methylation array-based platforms, such as the Illumina HumanMethylation450K and MethylationEPIC (850K) arrays, enable the generation of highly reproducible and cost-effective profiles across large cohorts and have been widely used for biomarker discovery and retrospective validation. Compared to bisulfite sequencing and EM-seq, methylation arrays offer lower genomic coverage because they analyze predefined CpG sites; however, they provide superior scalability, reproducibility, and cost-effectiveness, making them particularly well-suited for large retrospective cohorts and biomarker validation studies. Compared to sequencing-based approaches, methylation arrays offer greater scalability and lower costs for studies of large cohorts, but their predefined CpG coverage limits the identification of new methylation alterations and reduces flexibility across different tumor types and clinical scenarios (92, 93).

From a translational perspective, no single platform currently meets all the requirements for clinical implementation. Overall, bisulfite sequencing and EM-seq remain the preferred strategies for comprehensive methylome analysis and biological characterization, while methylation arrays serve as scalable platforms for retrospective profiling and validation. In contrast, targeted multi-CpG methylation assays currently offer the best balance between analytical sensitivity, reproducibility, cost-effectiveness, and clinical scalability, representing the most promising approach for clinical translation. Genomic-level approaches continue to be superior for biomarker identification, molecular stratification, and prediction of tissue of origin, while targeted multi-CpG methylation assays currently offer the best balance between analytical sensitivity, reproducibility, cost-effectiveness, and clinical scalability. In fact, large-scale studies based on circulating methylation signatures have demonstrated high diagnostic accuracy for the early diagnosis of cancer and improved performance compared to approaches based on ctDNA mutations, particularly in settings with a low tumor fraction (16, 46, 94). However, compared with their analytical performance in research settings, clinical implementation of methylation-based assays remains dependent on harmonization of pre-analytical variables, standardization of analytical workflows, definition of quality control criteria, and prospective validation in multicenter clinical trials (95–97).

## 4 Early detection and screening

### 4.1 Clinical need and positioning of epigenetic LB

Low-dose CT (LDCT) screening has transformed LC early detection and is currently recommended by international guidelines, including NCCN and ESMO, for high-risk individuals. A significant reduction in LC mortality of approximately 20-24%, primarily through a stage shift toward early, has been seen screening NSCLC patients by LDCT. In particular, current guidelines endorse LDCT screening in high-risk populations, typically current or former smokers with significant pack-year exposure. Despite these clear benefits, LDCT screening remains limited due to false-positive findings and overdiagnosis (4, 98–100), (**Table 2**).

**Table 2:** Liquid-biopsy DNA methylation studies for early lung cancer detection and classification of CT-detected pulmonary nodules.

Study	Cohort	Biofluid / strategy	Sensitivity / specificity / AUC	Key findings
**Lung EpiCheck (NCT02373917)** (107)	European training: 102 lung cancers (LC) + 265 controls; European validation: 179 + 137; Chinese validation: 30 + 15.	Plasma cfDNA; six-marker PCR assay with low (LCO) and high (HCO) cut-offs	AUC: 0.882 (Europe); 0.899 (China). Europe: LCO, 87.2%/64.2%; HCO, 74.3%/90.5%. China: LCO, 76.7%/93.3%; HCO, 56.7%/100%. Stage I sensitivity: Europe, 78.4% (LCO) and 62.2% (HCO); China, 70.0% (LCO) and 30.0% (HCO).	Cross-geographic validation and clear threshold trade-off. Case-control design; Chinese cohort was small; prospective screening validation remains necessary
***S. Hu* et al.** (105)	317 (198 tissue / 119 plasma); Plasma cohort n=106 (26 LC, 30 benign lesions, 50 healthy).	Plasma cfDNA; targeted bisulfite sequencing and seven differentially methylated regions (DMRs)	Plasma: sensitivity 81%; specificity 98%; AUC 0.94 (95% CI 0.86–1.00); accuracy 93%. Tissue AUC ≈0.96–0.97	High specificity for early cancer/nodule discrimination, but only 26 lung cancers and no stage-resolved operating characteristics
***Z. Wang* et al.** (108)	Model development n=513; independent validation n=172 (64 LC, 45 benign pulmonary disease, 63 healthy).	Plasma cfDNA; multiplex qPCR, 11-marker LunaCAM-S screening model and LunaCAM-D diagnostic model; sequential strategy	Validation: LunaCAM-S, LC vs healthy: sensitivity 90.6%, specificity 61.9%, AUC 0.90. LunaCAM-D, LC vs benign disease: sensitivity 51.6%, specificity 93.3%, AUC 0.81. Stage I sensitivity in the pooled cohort: 87.0% and 49.1%, respectively. LunaCAM-D + CEA: AUC 0.86.	The two models were developed for distinct clinical uses: LunaCAM-S as a sensitivity-oriented screening test and LunaCAM-D as a specificity-oriented diagnostic aid. Sequential application corrected 83.3% of the non-cancer cases misclassified by LunaCAM-S while retaining 55.2% of LC cases as positive. Prospective validation is required
***L. Ke* et al.** (106)	261 participants: 179 LC and 82 healthy controls. Training set: n=184 (126 LC, 58 controls); internal hold-out validation set: n=77 (53 LC, 24 controls)	Plasma cfDNA concentration combined with qPCR-based methylation analysis of *SHOX2, PTGER4*, *RASSF1A* and *H4C6*	Training AUC: 0.8012. Validation: sensitivity 77.36%; specificity 91.67%; AUC 0.8436 (95% CI 0.7565–0.9306).	The five-feature model outperformed the individual biomarkers and the three-feature model (training AUC 0.7875). Only *SHOX2* and *PTGER4* differed significantly between LC and controls, and model scores were lower in early-stage disease
***C. Du* et al.** (118)	237 participants enrolled; 202 successfully analyzed: 149 squamous LC (72 mass-type and 77 GGN-type cancers), 5 benign nodules and 48 healthy controls. Among 135 cases with available stage, 91 were stage I, 28 stage II, 15 stage III and 1 stage IV	Plasma cfDNA analyzed using three multiplex qMSP assays for *TAC1, CDO1, HOXA9, ZFP42, SOX17, RASSF1A* and *SHOX2*	LC vs pooled benign/healthy controls: sensitivity 86.7%; specificity 81.4%; AUC 0.891. GGN-type vs mass-type LC: sensitivity 77.1%; specificity 65.8%; AUC 0.753.	Good apparent discrimination, but the benign comparator included only five cases and no independent validation was performed
***B. Wei* et al.** (32)	500 participants. Training: n=351 (154 LC; 43 healthy controls, 145 benign lung diseases and 9 non-lung cancers). Independent cohort: n=149 (60 LC; 20 healthy controls, 62 benign lung diseases and 7 non-lung cancers	Plasma cfDNA qPCR; three-gene methylation panel: *SHOX2, RASSF1A* and *PTGER4*	Training: sensitivity 87.0%; specificity 98.0%; AUC 0.938 (95% CI 0.907–0.961). Validation: sensitivity 83.3%; specificity 94.4%; AUC 0.910. Training stage-specific sensitivity: stage I, 82.5% (33/40); stage II, 90.5% (19/21)	Robust three-marker performance with independent temporal validation; stage-specific sensitivities were not confirmed in validation and the study was single-center
***E. Alemany-Cosme* et al.** (103)	Prospective study with two independent cohorts. Discovery: 25 LC (stage I, n=4; II, n=5; III, n=6; IV, n=8; unknown, n=2) and 8 controls with nonmalignant respiratory diseases. Validation: 23 LC (stage I, n=3; II, n=3; III, n=7; IV, n=7; unknown, n=3) and 24 controls with pulmonary or non-pulmonary conditions	Plasma cfDNA genome-wide EPIC array; elastic-net 21-CpG episignature; *EMP2/IKZF2* assessed by ddPCR.	21-CpG episignature: internally cross-validated AUC 0.70. EMP2 independent validation: AUC 0.75 (95% CI 0.61–0.89). Sensitivity and specificity at a prespecified operating threshold: NR	The 21-CpG signature was predominantly hypomethylated (18/21 loci) but was not validated as a complete panel. *EMP2* promoter hypomethylation was confirmed using an independent platform and cohort, whereas *IKZF2* did not validate
***L. Li* et al.** (109)	100 tissue samples (56 malignant, 44 benign) and 146 plasma samples (89 malignant, 57 benign) from 214 participants; 48 provided paired tissue and plasma. Stage I represented 73.2% of tissue cancers and 69.7% of plasma cancers; stage II represented 8.9% and 9.0%, respectively. Tissue and plasma cohorts were each randomly divided 7:3 into training and internal validation sets.	Plasma cfDNA, Marker selection was performed separately in tissue and plasma, generating a six-marker tissue model and a nine-CpG plasma	Tissue: training, sensitivity 90%, specificity 97%, AUC 0.988; validation, 92%/94%/0.977. Plasma: training, 98%/100%/0.998; validation, 81%/59%/0.791.	The plasma model showed moderate discrimination in internal validation, with a marked decline from training. No plasma CpG met FDR <0.05; marker selection therefore used a relaxed threshold of FDR <0.20. The small validation set (27 LC and 17 benign cases), low specificity and absence of external validation raise concerns regarding model instability and overfitting
***BMM. Wever* et al.** (114)	Prospective single-center, non-screening cohort: 46 surgically treated nonmetastatic NSCLC patients enrolled, of whom 44 had valid results, and 50 age- and sex-matched healthy controls. Analyzed cases included stage I, n=28; stage II, n=4; and stage III, n=12	Urine cfDNA methylation-specific PCR; *CDO1, SOX17* and *TAC1*.	Non-cross-validated CDO1 + SOX17 model: sensitivity 57%; specificity 86%; AUC 0.78 (95% CI 0.68–0.87). Leave-one-out cross-validation: sensitivity 55%; specificity 86%; AUC 0.71.	*CDO1* and *SOX17* methylation was significantly increased in NSCLC urine, whereas *TAC1* was not. The study demonstrates technical feasibility, but moderate sensitivity, absence of independent validation, healthy-only controls and substantial smoking imbalance between cases and controls limit screening applicability
***T. Powrózek* et al.** (102)	Pilot case-control study: 70 untreated LC (55 NSCLC and 15 SCLC) and 80 healthy controls. NSCLC stages: I, n=8; II, n=12; IIIA, n=10; IIIB, n=9; IV, n=16. The grouped potentially operable stage I–IIIA subgroup included 30 patients; all SCLC cases had advanced disease	Bisulfite-converted plasma cfDNA; quantitative methylation-specific real-time PCR targeting *RTEL1* and *PCDHGB6*	Individual markers: *RTEL1*, sensitivity 51.4% and specificity 91.2%; *PCDHGB6*, 41.4% and 98.7%. Combined two-marker test: sensitivity 62.9%; specificity 90.0%; AUC 0.755 (95% CI 0.678–0.838). Stage I–IIIA NSCLC: reported sensitivity 64.6%; specificity 88.4%; AUC 0.752 (95% CI 0.649–0.837)	The two-marker strategy improved sensitivity compared with either marker alone while retaining relatively high specificity. However, this was a small exploratory study using healthy controls only, with no independent validation or benign pulmonary comparators
***W. Liang* et al. (PulmoSeek)** (119)	Multicenter retrospective Chinese cohort: 585 patients with CT/LDCT-detected pulmonary nodules enrolled; 529 analyzed (413 malignant, 116 benign). Model development n=389, including training n=309 and test n=80; held-out validation n=140 (100 malignant, 40 benign). Predominantly stage I–II disease	Plasma cfDNA targeted bisulfite methylation sequencing; panel covering 12,899 regions and 105,844 CpGs; final 100-feature PulmoSeek model	Validation: sensitivity 99.0%; specificity 32.5%; AUC 0.843 (95% CI 0.769–0.918); accuracy 80.0%. Stage I, validation: stage IA 94.1% and stage IB 100% sensitivity. 6–20 mm nodules, validation (n=103): sensitivity 100%; specificity 30.0%; AUC 0.844. Combined test + validation: stage 0–I sensitivity 97.1%	Very high sensitivity supports potential use as a rule-out test for CT-detected nodules, but specificity was low. The validation set was a retrospective hold-out from the same multicenter cohort, with pathology-confirmed cases and a high malignancy prevalence
***W. Liang* et al.** (111)	Tissue development cohort n=230 (129 malignant, 101 benign) and independent tissue validation cohort n=58. Plasma pulmonary-nodule cohort n=132, randomly divided into training n=66 (40 malignant, 26 benign) and held-out test n=66 (39 malignant, 27 benign); additional 118 age- and sex-matched asymptomatic normal controls. Nodules were <3 cm and surgically confirmed	Plasma cfDNA targeted bisulfite methylation sequencing using the AnchorIRIS assay	Held-out plasma test: sensitivity 79.5% (95% CI 63.5–90.7%); specificity 85.2% (66.3–95.8%); AUC 0.816 (0.703–0.929). Stage IA: sensitivity 75.0% (15/20); stage IB: 85.7% (6/7). Only one stage IIA case was included and was classified as negative. Specificity versus asymptomatic normal controls: 93.2% (89.0–98.3%)	Proof-of-concept for distinguishing malignant from benign CT-detected nodules <3 cm, with moderate sensitivity in stage IA–IB disease
***W. Huang* et al.** (120)	Primary cohort: 104 LC (stage I n=48; II n=15; III n=20; IV n=21) and 36 benign lung diseases; 50 normal controls were included in descriptive comparisons. External validation: 19 LC (stage I n=12; II n=4; III n=3) and 11 benign lung diseases	Plasma cfDNA qPCR measurement of *SHOX2* and *PTGER4* methylation; total methylation (TM = *SHOX2* + *PTGER4*) integrated with plasma CYFRA21–1 in a two-step decision tree	Primary decision tree: sensitivity 85.6%; specificity 100%; AUC 0.921. External validation: reported sensitivity 78.5%; specificity 90.9%; AUC 0.849. Primary methylation alone: *SHOX2* 60.6%/97.1%/0.851; *PTGER4* 53.8%/97.1%/0.847; TM 65.4%/97.1%/0.861	Integrating methylation with CYFRA21–1 increased sensitivity and discrimination compared with methylation alone in the primary cohort
***C. Chen* et al.** (112)	Single-center surgical cohort of 246 patients with CT-detected nodules ≤3 cm: 163 stage I NSCLC (T1N0 n=102; T2N0 n=61) and 83 pathologically confirmed benign lesions; an additional 20 healthy volunteers were included in descriptive methylation analyses	Plasma cfDNA modified MOB-qMSP evaluating eight genes. Main three-gene panel: *CDO1/SOX17/HOXA7*; alternative *CDO1/TAC1/SOX17* panel evaluated for subcentimeter nodules. Clinical integration included age, pack-years, COPD status, nodule size and pulmonary-function parameters	Main panel: sensitivity 90%; specificity 71%; AUC 0.88 (95% CI 0.84–0.93). Main panel + clinical predictors: 91%/79%/0.94 (0.91–0.96). By size, main panel: 2.1–3.0 cm, 91%/90%/0.95; 1.1–2.0 cm, 74%/93%/0.92; ≤1.0 cm, 64%/82%/0.75. Alternative panel in ≤1.0-cm nodules: 71%/82%/0.81	Entire malignant cohort consisted of stage I, small, node-negative NSCLC, demonstrating feasibility in very early disease. Performance declined markedly with decreasing nodule size, although the alternative panel partially improved subcentimeter sensitivity. Results were derived and evaluated in the same resection-selected cohort, without internal or external validation; stage II performance was not assessed
***Liang* et al.** (113)	963 participants with solitary, noncalcified 5–30-mm nodules from two prospectively collected multicenter Chinese cohorts. Development cohort n=620 from 10 hospitals: training n=341 and site-separated internal validation n=279. Independent external validation n=343 from 18 hospitals. Overall, 661 malignant and 302 benign nodules; 93.8% of malignancies were stage 0–I, whereas only 14 were stage II	Plasma cfDNA targeted methylation sequencing using a customized 263-DMR panel; final 40-marker logistic-regression methylation score integrated with age and five CT features: long diameter, short diameter, their product, nodule density type and mean attenuation value	Methylation-only model: AUC 0.81 in both internal and external validation. Combined model: internal-validation AUC 0.90 (95% CI 0.86–0.94); external-validation AUC 0.89 (0.85–0.92). Pooled validation two-threshold strategy: low threshold 0.22, rule-out sensitivity 98.1% and NPV 91.8%; high threshold 0.94, rule-in specificity 90.4% and PPV 93.9%	The clinically integrated model was independently validated and outperformed methylation alone. However, rule-out and rule-in performance came from different thresholds that were derived and assessed in the pooled validation cohorts

AUC, area under the receiver operating characteristic curve; CEA, carcinoembryonic antigen; cfDNA, cell-free DNA; CI, confidence interval; COPD, chronic obstructive pulmonary disease; CpG, cytosine-phosphate-guanine dinucleotide; CT, computed tomography; CYFRA21-1, cytokeratin 19 fragment; ddPCR, droplet digital polymerase chain reaction; DMR, differentially methylated region; FDR, false discovery rate; gDNA, genomic DNA; GGN, ground-glass nodule; HCO, high cut-off; LC, lung cancer; LCO, low cut-off; LDCT, low-dose computed tomography; MOB-qMSP, methylation-on-beads quantitative methylation-specific polymerase chain reaction; NPV, negative predictive value; NR, not reported; NSCLC, non-small cell lung cancer; PCR, polymerase chain reaction; PPV, positive predictive value; qMSP, quantitative methylation-specific polymerase chain reaction; qPCR, quantitative polymerase chain reaction; SCLC, small cell lung cancer; TM, total methylation. The studies and author names indicated in bold correspond to the respective references listed in the reference section.

Epigenetic LB assays emerged as a promising adjunct to imaging. Unlike mutation-based approaches, DNA methylation alterations occur in the early stages of carcinogenesis and can generate robust tumor-specific signals even when the amount of circulating tumor DNA is limited. This advantage stems from the fact that malignant transformation is accompanied by widespread and coordinated epigenetic reprogramming, which includes promoter hypermethylation, global hypomethylation, and lineage-specific methylation changes that simultaneously affect multiple genomic regions and CpG loci. Consequently, methylation-based tests can capitalize on the cumulative tumor-associated signals distributed across numerous CpG loci, increasing the likelihood of detection in early-stage tumors characterized by low ctDNA release. In contrast, mutation-based ctDNA tests are inherently dependent on the presence and accurate detection of specific patient-specific variants, which can become challenging when the number of circulating mutant molecules falls below the analytical detection limit (45, 101). Therefore, methylation-based tests capture patterns of coordinated epigenetic alterations associated with tumor identity and cellular origin, rather than relying solely on the detection of individual tumor-specific mutations. Furthermore, DNA methylation signatures are highly tissue- and lineage-specific, allowing for the simultaneous detection of tumor-derived cfDNA and the inference of its tissue of origin (16, 45). It is important to note that methylation-based approaches can also reduce confounding effects arising from clonal hematopoiesis, a significant limitation of mutation-based ctDNA tests (101). These features position cfDNA methylation as a complementary tool of LDCT, with potential roles in pre-screening risk enrichment, post-LDCT triage of indeterminate nodules, and improving patient selection for further diagnostic work-up (63).

A critical distinction must be made between tumor-derived cfDNA methylation and whole-blood (germline) methylation signatures. While the latter, can provide objective risk stratification with performance comparable to clinical models (AUC ~0.73), they primarily reflect cumulative environmental exposure rather than tumor presence and do not significantly improve existing prediction tools. In contrast, cfDNA methylation captures tumor-derived epigenetic alterations and is therefore more suitable for early cancer detection (100).

### 4.2 Clinical performance of cfDNA methylation assays: from single biomarkers to multi-gene panels

The clinical performance of cfDNA methylation assays for early LC detection has evolved over the past decade, transitioning from single-gene biomarkers to multi-gene panels. Early studies focused on the detection of promoter methylation in individual tumor suppressor genes, demonstrating proof-of-concept for epigenetic analysis of LB but also highlighting intrinsic limitations in terms of clinical sensitivity. In a pilot study (n=70 patients and 80 controls), *Regulator of Telomere Elongation Helicase 1 (RTEL1)* and *Protocadherin Gamma Subfamily B 6 (PCDHGB6)* methylation showed high specificity (up to 98.7%) but moderate sensitivity (~40–50%), Importantly, combining these markers led to an improvement in performance (AUC ~0.75), establishing an early rationale for multi-marker approaches (102).

Parallel efforts using genome-wide methylation profiling have reinforced this concept. Unbiased analyses of cfDNA methylomes have identified DMRs and epi-signatures discriminating LC from controls. For example, a 21-CpG epi-signature derived from genome-wide analysis highlighted moderate diagnostic accuracy (AUC ~0.70–0.75), with subsequent validation of specific loci such as *Epithelial Membrane Protein 2 (EMP2) (*103*)*. Similarly, integrative approaches combining cfDNA methylation with tissue-derived datasets have identified multi-CpG panels with strong diagnostic potential, such as a 7-CpG signature associated with lung adenocarcinoma risk and prognosis (104). However, these discovery-driven studies consistently underscore a key limitation: single loci or small signatures, while biologically informative, rarely achieve sufficient performance for clinical use.

The shift toward targeted multi-gene panels has markedly improved the diagnostic accuracy. Combining multiple methylation markers significantly enhancement both sensitivity and specificity compared to single-gene approaches. A three-gene panel (*SHOX2, RASSF1A, PTGER4*) achieved AUC values exceeding 0.90, with sensitivity around 83–87% and specificity up to 98%, including robust detection of early-stage disease (32). These findings consistently demonstrated that integrative multiple CpG targets improves signal-to-noise ratio, particularly in low tumor burden settings.

More advanced targeted sequencing approaches have further refined panel-based strategies. High-throughput analyses of thousands of CpG sites enabled the identification of DMR panels with excellent diagnostic performance. A seven-DMR model derived from over 9,000 loci achieved AUC values of ~0.94 in plasma, with high specificity (up to 98%) for distinguishing malignant from benign pulmonary lesions (105).

Recent studies have explored the integration of quantitative and qualitative cfDNA features to further enhance diagnostic performance. A combining model of methylation markers and cfDNA concentration improved accuracy (AUC ~0.84) in comparison to individual biomarkers, highlighting the complementary value of tumor burden–related metrics (106).

While genome-wide sequencing approaches offer high sensitivity and discovery potential, they are often limited by cost and technical complexity. In contrast, PCR-based assays are more scalable and clinically feasible alternative. The Lung EpiCheck test, based on a six-marker methylation panel, achieved AUC values of ~0.88–0.90 across validation cohorts (overall n≈700 subjects) detecting a substantial proportion of stage I tumors (107). Similarly, the LunaCAM assay employs a multiplex qPCR platform targeting 11 methylation markers and introduces a dual-model strategy, consisting of a high-sensitivity screening model (LunaCAM-S) followed by a high-specificity diagnostic model (LunaCAM-D). This sequential approach posed basis for identifying potential cancer cases reducing false positives (sensitivity ~90%) in early-stages (108). Droplet digital PCR (ddPCR)-based multiplex assays support the feasibility of cost-effective and sensitive detection, although sensitivity remains dependent on tumor stage and ctDNA shedding.

Despite these advances, important limitations persist. Variability in study design, cohort composition, and the referenced markers contributes to heterogeneity in technical performance. Tissue–plasma concordance analyses confirm that cfDNA methylation reflects tumor biology, but quantitative discrepancies remain, particularly in early-stage settings (109). Overall, the clinical performance of cfDNA methylation assays has progressively improved through the transition from single-gene biomarkers to multi-gene panels and integrated models. While current assays demonstrate promising accuracy for early detection, particularly when using targeted panels and scalable platforms, their ultimate clinical utility depends on further validation in prospective screening settings and integration into diagnostic workflows.

### 4.3 Clinical applications of cfDNA methylation: pulmonary nodules and emerging uses

cfDNA methylation showed promising results evaluating pulmonary nodules and distinguishing between benign and malignant lesions. Unfortunately, the majority of nodules are benign, leading to substantial overdiagnosis, repeated imaging, and unnecessary invasive procedures (110). Several studies have demonstrated that plasma cfDNA methylation profiling can achieve clinically meaningful diagnostic accuracy in this challenging scenario. Targeted methylation sequencing approaches built on a limited number of markers, evaluated 132 patients with pulmonary nodules plus 118 healthy controls achieving sensitivities of ~75–80% and specificities of ~85% (AUC ~0.82) for early-stage LC, with consistent performance across different nodule types, including solid and subsolid lesions (111). Importantly, such assays retain diagnostic capability even in stage I disease, although sensitivity decreases in very small tumors due to low ctDNA shedding. In studies specifically focusing on small nodules (≤3 cm), multi-gene methylation panels highlighted sensitivities up to ~90% with specificities around 70–80% (AUC ~0.88), while performance declined for sub-centimeters lesions (sensitivity ~60–65%) (112),. More advanced models based on high-dimensional methylation signatures and ML meliorated diagnostic performance. Notably, a plasma methylation model developed and validated in LDCT-positive cohorts (n=529 analyzed patients, including an independent validation set of n=140) achieved an AUC of 0.84 with near-perfect sensitivity (~99%) and negative predictive value (~99.7%) in LDCT-positive populations, enabling reliable exclusion of malignancy in indeterminate nodules. From a clinical perspective, these characteristics support the use of cfDNA methylation assays as rule-out tests within LDCT-based screening workflows, with the potential to significantly reduce unnecessary biopsies and surgical interventions, particularly in patients with low- to intermediate-risk nodules (113).

Beyond plasma-based assays, methylation analysis is being explored in alternative biofluids, expanding the concept of non-invasive cancer detection. Urine-based cfDNA methylation has demonstrated feasibility for detecting non-metastatic LC, with moderate sensitivity (~55%) but good specificity (~86%), suggesting a potential role as an adjunct screening tool (114). Similarly, methylation analysis of bronchial washing fluid in a diagnostic cohort (n=187 patients) has shown improved diagnostic sensitivity compared to cytology alone (approximately 77–81% vs ~39%), with specificity around 90%, particularly when combined with conventional diagnostic approaches, although its applicability remains limited by the need for invasive procedures (103, 115).

Finally, cfDNA methylation profiling may extend beyond detection to tumor characterization. Recent studies have demonstrated the ability of methylome-based approaches to non-invasively distinguish LC subtypes, such as adenocarcinoma and squamous cell carcinoma, with good accuracy, particularly in advanced disease (71). This highlights the potential of epigenetic LB to evolve from a purely diagnostic tool toward a broader role in precision oncology, although such applications remain exploratory in early-stage settings.

However, the available evidence does not establish comparable diagnostic performance across lung adenocarcinoma (LUAD), lung squamous cell carcinoma (LUSC/SQCC), and small-cell lung cancer (SCLC). Most early-detection cohorts were dominated by LUAD and did not report histology-stratified sensitivity, specificity, or AUC values, whereas one mixed-histology study found higher methylation-based scores in LUSC than in LUAD, suggesting a potential histology-dependent signal. In histology-specific studies, a three-marker LUSC panel achieved an AUC of 0.972, with 100% sensitivity and 79.6% specificity against healthy controls, while a genome-wide SCLC methylation classifier detected 93% of limited-stage and 100% of extensive-stage cases, with corresponding AUROCs of 0.986 and 1.00. Nevertheless, these estimates were derived from small, selected, and non-comparable cohorts and lacked direct cross-histology validation (116, 117). A separate methylome-based classifier distinguished LUAD from LUSC with an AUC of 0.808 in the development cohort and 0.747 in independent validation, with lower performance in stage I-II than in stage III-IV disease (AUC 0.729 vs 0.867, respectively) (71),. These findings support the biological relevance of histology-specific methylation profiles and the potential of epigenetic liquid biopsy for non-invasive tumor subtyping. However, assay performance cannot currently be assumed to be equivalent across histological subtypes, and prospective head-to-head validation in early-stage and clinically representative populations remains necessary.

## 5 Minimal residual disease and post-surgical surveillance

MRD refers to the presence of tumor-derived signals that persists after curative-intent treatment in patients with no radiological evidence of disease (121). In early-stage and locally advanced NSCLC, MRD detection emerged as a critical unmet need involving substantial proportion of patients relapsing after surgical resection. The ability to identify residual disease shortly after treatment offers a unique opportunity to refine risk stratification and personalize postoperative management, including the selection of adjuvant or consolidation therapies (122).

ctDNA analysis has become the most extensively investigated approach for MRD detection, supported by a large and growing body of evidence demonstrating its strong prognostic value in early-stage NSCLC (123). Notably, ctDNA-based MRD positivity is associated with an extremely high risk of recurrence, with more than 90–95% of patients relapsing in the absence of further treatment, while ctDNA clearance correlates with improved outcomes. However, the detection of MRD remains technically challenging, as ctDNA levels are often exceedingly low in early-stage disease, frequently below 0.01-0.1% of total cfDNA, resulting in limited sensitivity and a substantial rate of false-negative results (124, 125).

In this context, two complementary methodological paradigms have emerged: tumor-informed approaches, which rely on patient-specific molecular profiling, and tumor-agnostic approaches, which use predefined genomic or epigenomic signatures, including DNA methylation. These strategies differ in sensitivity, specificity, and clinical scalability, and their integration, together with longitudinal monitoring, represents a promising direction to improve MRD detection and guide post-surgical surveillance (125–127).

### 5.1 Tumor-informed methylation MRD approaches

Tumor-informed methylation-based MRD (timMRD) approaches represent one of the most promising strategies for improving the sensitivity of postoperative disease monitoring in LC. These approaches rely on the identification of patient-specific methylation patterns from resected tumor tissue longitudinally tracking tumor private hallmarks. Compared with mutation-based MRD assays, methylation-based approaches can interrogate a larger number of tumor-specific alterations simultaneously, thereby increasing the probability of detecting residual disease when only minimal amounts of tumor-derived cfDNA are present (89, 90). This advantage stems from the biological nature of cancer-associated methylation alterations, which often involve coordinated changes across multiple CpG sites and genomic regions rather than isolated molecular events. Therefore, methylation profiling can generate a broader and more redundant tumor-derived signal, reducing the impact of limitations related to stochastic sampling that could affect the detection of individual somatic mutations in conditions characterized by very low levels of ctDNA This feature is particularly relevant following curative surgery, where the limited abundance of circulating tumor DNA can reduce the sensitivity of mutation-based approaches.

In a large longitudinal study including 195 patients with NSCLC (128), timMRD demonstrated superior sensitivity compared to mutation-based ctDNA analysis (90.9% vs 45.5%), with significantly improved diagnostic accuracy (AUC 0.87 vs 0.68). Importantly, postoperative timMRD positivity was strongly associated with worse disease-free survival (HR 3.08), and dynamic changes in methylation signal reflected tumor burden, decreasing after surgery and increasing prior to clinical relapse. Notably, methylation-based MRD detection can predict radiological recurrence up to 10 months (~303 days).

In a cohort of 65 patients, methylation-based MRD detection was achievable in up to 96% of patients at baseline then confirmed in 70% of post-operative samples, outperforming mutation-based detection rates, which dropped to ~23-25% in the postoperative setting (129). Importantly, methylation signals identified recurrence even in patients with undetectable ctDNA mutations, highlighting their complementary role and increased sensitivity in low tumor burden conditions.

Emerging data suggest that the biological source of cfDNA may significantly influence MRD detection and its clinical interpretation. In a smaller exploratory cohort (n=43), postoperative pleural fluid analysis demonstrated higher ctDNA levels compared to matched plasma samples, with significantly increased mean variant allele frequency (p=0.029), indicating improved sensitivity for detecting locoregional disease. Notably, pleural fluid–based MRD detection identified 100% of locoregional relapses in this cohort and was associated with high-risk pathological features, including nodal involvement (N1-N2), visceral pleural invasion, and airway invasion (130).

Despite these encouraging results, timMRD approaches remain limited by their dependence on tumor tissue availability, increased analytical complexity, and reduced scalability. Nevertheless, their high sensitivity and ability to detect recurrence months before imaging position them as a powerful tool for personalized postoperative surveillance.

### 5.2 Tumor-agnostic methylation MRD approaches

Tumor-agnostic methylation-based MRD approaches represent an emerging and clinically attractive strategy for postoperative surveillance in LC. These assays rely on predefined panels of cancer-specific DMRs, enabling detection of tumor-derived epigenetic signals. Compared to tumor-informed approaches, their main advantage lies in scalability, faster turnaround time, and broader applicability across patient populations, particularly in settings where tumor tissue is unavailable or insufficient (131).

Analytical validation studies demonstrated that tumor-agnostic methylation assays can achieve remarkably low limits of detection. For example, the STELLA assay, based on a panel of 341 hypermethylated regions, was able to detect tumor fractions as low as ~0.02% (limit of detection ≈0.019%), approaching the sensitivity of advanced mutation-based MRD assays (132). In comparative analyses with reference mutation-based assay (PROPHET), STELLA showed good concordance (R = 0.73, p<0.001) and high specificity (~97.9–98%), although sensitivity varied depending on the clinical context. Notably, sensitivity reached ~73.8% in enriched cohorts but decreased to ~41.4% in postoperative MRD settings, highlighting the challenges of detecting extremely low tumor burden after curative-intent treatment.

Despite these limitations, tumor-agnostic methylation assays have demonstrated clinically meaningful prognostic value. In postoperative cohorts, MRD positivity using methylation-based assays was strongly associated with recurrence risk (HR ~3.98, p<0.001), supporting their role as prognostic biomarkers in early-stage disease (132). In a cohort of 78 patients under serial monitoring, tumor-naïve methylation-based MRD detection predicted both recurrence-free survival (HR up to 5.20) and overall survival, with ctDNA positivity preceding radiological relapse of approximately 5.4 months. Importantly, diagnostic performance in this setting was moderate, with sensitivity of ~63.6% and specificity of ~78.1%, reflecting the inherent trade-off between sensitivity and scalability (133).

An important advance of tumor-agnostic approaches can also provide quantitative and dynamic information. Methylation-based assays have been shown to measure tumor fraction, such as the differentially methylated allele fraction (DMAF), which strongly correlate with mutation-based variant allele fractions (ρ≈0.83 in LC cohorts) (134),. This quantitative capability enables longitudinal monitoring of disease dynamics and may support more refined risk stratification and treatment decision-making.

Furthermore, emerging evidence highlights the importance of dynamic changes in methylation signals rather than single timepoint measurements. In a prospective phase II study including 60 patients treated with neoadjuvant chemoimmunotherapy, early reductions in methylation-derived metrics such as the methylated fragment ratio (MFR) were strongly associated with major pathological response (74.5% vs 11.1% in patients with persistently high levels), whereas baseline measurements showed limited predictive value (135). These findings suggest that tumor-agnostic methylation assays may serve as real-time biomarkers of treatment response and residual disease, paralleling the concept of MRD clearance in mutation-based assays.

However, not all tumor-agnostic methylation approaches demonstrated robust performance in early-stage disease. In a large retrospective cohort of stage I 260 patients NSCLC, preoperative methylation-based ctDNA detection did not significantly predict long-term relapse, although it showed an association with early recurrence and pathological upstaging, suggesting a role in identifying biologically aggressive disease rather than true MRD (136). Similarly, a large real-world cohort (n=895) demonstrated that detection rates are strongly stage-dependent, with sensitivity as low as ~31% in stage I disease (and as low as 13% in lung adenocarcinoma) compared to ~71% in stage II. Importantly, ctDNA positivity in stage I lung adenocarcinoma was significantly associated with worse outcomes, with a 2-year recurrence-free survival of 69% versus 91% in ctDNA-negative patients. From a clinical perspective, these findings indicate that tumor-agnostic methylation assays in early-stage settings are more reflective of tumor burden and biological aggressiveness than of MRD and may therefore be better positioned as risk stratification tools, particularly for identifying patients with occult higher-stage disease or candidates for intensified perioperative treatment assays (137).

To overcome these limitations, recent studies have explored integrated tumor-agnostic approaches combining methylation with additional biological and clinical features. A multi-parametric model incorporating cfDNA methylation, somatic mutations, and radiological tumor volume demonstrated improved prognostic performance, with ctDNA persistence emerging as a strong predictor of relapse (RFS HR ~4.0; OS HR up to ~16.5) and enabling stratification of patients into distinct risk groups. These findings underscore the importance of integrating epigenetic signals with complementary biomarkers to enhance MRD detection (138).

Overall, tumor-agnostic methylation MRD approaches offer a scalable and clinically feasible alternative to tumor-informed assays, with high specificity and demonstrated prognostic relevance, summarized in **Table 3**. While tumor-informed approaches generally achieve higher sensitivity and longer lead times for recurrence detection, tumor-agnostic strategies avoid the need for prior tumor tissue profiling and patient-specific assay development, facilitating broader clinical implementation. Their optimal clinical role likely lies in combination with other biomarkers and longitudinal monitoring strategies.

**Table 3:** Methylation-based approaches for MRD detection in LC.

Study	Approach	Setting	Cohort	Biofluid / Strategy	Detection rate	Sensitivity/ Specificity	Key finding
MEDAL study (NCT03634826) (128)	Tumor-informed	Post-surgery	Medal cohort (n=195), Dynamic validation cohort (n=36)	Plasma / methylation	NR	90.9% (vs 45.5% mutations) / ~67% (vs ~90% mutations)	AUC (0.87 vs 0.68); Higher timMRD-scores associated with shorter DFS in the MEDAL cohort (HR: 3.08, 95% CI: 1.48–6.42; P = 0.002) and DYNAMIC cohort (HR: 2.80, 95% CI: 0.96–8.20; P = 0.041)
*H. Li* et al. (129)	Tumor-informed	Post-surgery	65	Plasma / Mutation + methylation	~70–75% MRD positivity at post-operative timepoints	NR	Lead time before radiologic recurrence (0.5–7 months)
*N. Earland* et al. (130)	Tumor-informed	Post-surgery	43	Plasma vs pleural fluid / DMRs	100% detection of locoregional pleural fluid relapse	NR	Pleural fluid outperforms plasma for locoregional MRD
*Bo Yang* et al. (132)	Tumor-agnostic	Pre/post-surgery	148	Plasma / methylation (341 DMRs)	32.6% (pre-op); LOD 0.02%	PPA 73.8% / 41.4%; NPA ~98%	Ultra-low LOD (~0.02%), high specificity, moderate sensitivity
*J. Lee* et al. (133)	Tumor-agnostic	Post-treatment	78	Plasma / Methylation	NR	63.6% / 78.1%	Predicts recurrence and OS; MRD precedes radiologic relapse (mean lead time 5.4 months); real-world feasibility
RENAISSANCE study (NCT04606303) (135)	Tumor-agnostic	Neoadjuvant	60	Plasma / methylation (MFR) + aneuploidy (CAFF)	NR	NR	Dynamic MFR/CAFF changes predict MPR; low status at surgery strongly associated with response; integrated model AUC 0.86
*Y. Bossé* et al. (136)	Tumor-agnostic	Pre-surgery	260	Plasma-only methylation (MCED)	27-36% (stage I)	NR	Not predictive of 5-year relapse; associated with early relapse (≤2 years, adenocarcinoma) and pathological upstaging
*T. H. Hong* et al. (137)	Tumor-agnostic	Pre-surgery	895	Plasma / cfDNA methylation (MCED-like)	31% (stage I), 71% (stage II)	NR	Stage I ctDNA+ associated with worse RFS (similar to stage II); predicts upstaging and high-risk biology; limited prognostic value in non-LUAD
*P. Parsana* et al. (134)	Tumor-agnostic	Cross-sectional (multi-cancer validation)	113 (lung)	Plasma / methylation (DMAF quantification)	NR	NR	Strong correlation between methylation-based DMAF and tumor-informed VAF (ρ ≈ 0.83 in lung); consistent across subtypes and independent of clinical variables

MRD, Minimal Residual Disease; ctDNA, circulating tumor DNA; cfDNA, cell-free DNA; MCED, Multi-Cancer Early Detection; MFR, Methylation Fraction/Methylation Feature Representation; CAFF, Copy-number Alteration Fraction/Aneuploidy Fraction Feature; DMAF, Differentially Methylated Feature (or Fraction); DMRs, Differentially Methylated Regions; LOD, Limit of Detection; AUC, Area Under the Curve; PPA, Positive Percent Agreement; NPA, Negative Percent Agreement; HR, Hazard Ratio; CI, Confidence Interval; DFS, Disease-Free Survival; RFS, Recurrence-Free Survival; OS, Overall Survival; MPR, Major Pathological Response; NR, Not Reported.

### 5.3 Comparison with mutation-based ctDNA MRD approaches

Available evidence suggests that methylation- and mutation-based MRD assays have distinct and potentially complementary strengths. Compared with mutation-based assays, methylation profiling can simultaneously interrogate coordinated alterations across multiple CpG sites and genomic regions, generating a broader and potentially more redundant tumor-derived signal than the detection of individual somatic variants. This may reduce the impact of stochastic sampling and increase the probability of detecting residual disease at very low tumor fractions, a feature that is particularly relevant after curative-intent surgery, when tumor-derived cfDNA is extremely scarce. Methylation-based approaches may also provide tissue-of-origin information and enable scalable tumor-agnostic testing without prior tumor sequencing (128, 129, 139).

In the MEDAL study, tumor-informed methylation-based MRD achieved greater sensitivity than mutation-based ctDNA analysis in NSCLC (90.9% vs 45.5%) and a higher AUC (0.87 vs 0.68), although this gain was accompanied by lower specificity (approximately 67% vs 90%). In a real-world cohort of 78 patients with definitively treated NSCLC, a tissue-naïve methylation-based assay predicted recurrence with 63.6% sensitivity and 78.1% specificity and preceded radiological relapse by a mean of 5.4 months, supporting its prognostic feasibility but also showing that its current accuracy remains insufficient to replace established surveillance procedures (128).

Although conducted in a different disease setting, the OPTIMISE trial in oligometastatic colorectal cancer provides a direct technical comparison between tumor-agnostic methylation- and mutation-based ddPCR assays. The methylation assay detected ctDNA in 13.0% of longitudinal samples compared with 7.8% using *KRAS/NRAS/BRAF* mutations, with an overall concordance of 84%. Methylation identified additional positive samples when the tumor lacked a trackable mutation, whereas some mutation-only signals were not detected in the corresponding tissue and were considered potentially attributable to clonal hematopoiesis. Although these findings cannot be directly extrapolated to lung cancer, they illustrate the complementary and non-interchangeable information captured by the two approaches (140).

Overall, methylation-based MRD assays may offer greater sensitivity and scalability, whereas mutation-based approaches currently provide higher specificity, greater clinical maturity, and actionable genomic information. Therefore, the available evidence does not yet support the replacement of mutation-based MRD testing by methylation profiling. Their most promising clinical application may involve integrated or sequential strategies in which methylation is used for sensitive longitudinal surveillance and mutation-based sequencing is subsequently applied to confirm tumor-derived signals and identify therapeutically relevant alterations. Prospective head-to-head trials are required to determine whether these strategies improve postoperative treatment selection and clinical outcomes in NSCLC.

## 6 Integration and future directions

### 6.1 Multi-omics integration: methylation combined with mutations, fragmentomics and machine learning -driven approaches

Recent advances in LB investigations suggest that integrating cfDNA methylation profiles with additional molecular and computational features may considerably enhance the accuracy and specificity of LC detection compared with single-analyte approaches.

**Figure 2:**
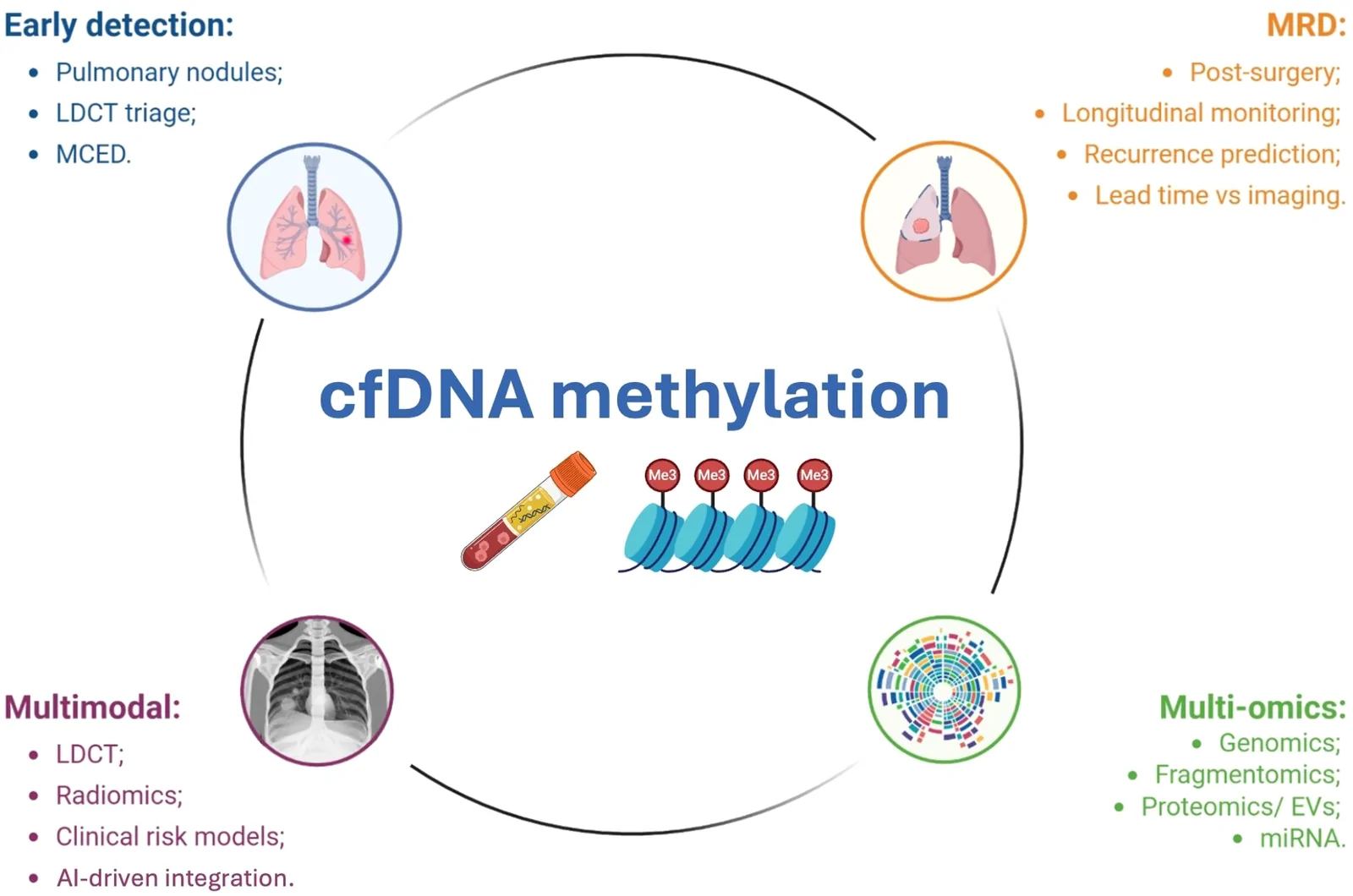
Integrating cfDNA methylation into multi-omics and multimodal frameworks for early cancer detection and MRD assessment. cfDNA methylation, positioned at the center, connects key clinical applications within a continuous evaluation framework. In early detection, it supports the assessment of pulmonary nodules, LDCT triage, and MCED strategies. In the MRD setting, it enables post-surgical assessment, longitudinal monitoring, and early prediction of recurrence, often providing lead time over conventional imaging. These applications are enhanced by multi-omics approaches, integrating methylation with genomics, fragmentomics, EVs, and miRNAs. In parallel, multimodal frameworks combine molecular data with imaging and clinical parameters, including LDCT, radiomics, and clinical risk models, supported by AI/ML for improved patient stratification and personalized management. AI/ML, artificial intelligence/machine learning; cfDNA, cell-free DNA; EVs, extracellular vesicles; LDCT, low-dose computed tomography; MCED, multi-cancer early detection; miRNAs, microRNAs; MRD, minimal residual disease.

Multi-omic strategies combine complementary molecular layers (**Figure 2**) as well as epigenetic and genomic alterations, fragmentomic signatures, in addition to EVs and miRNAs, together with AI-based analytical frameworks, to uncover different tumor-derived signals and boost biological characterization (141).

*Duan J* et al. investigated the performance of cfDNA methylation, somatic mutation profiling, and circulating protein markers in a prospective cohort of PROMISE study, including 1,706 patients with nine types of cancer, among which LC. Methylation-based classifiers demonstrated improved performance compared with models based exclusively on mutation or protein signatures, supporting that cfDNA methylation represent a key source of information in blood-based cancer detection strategies. The integration of multiple analytical layers achieved sensitivity and specificity values of 75% and 98%, respectively. Nevertheless, it is important to note that sensitivity remained limited in the early stages of the disease, particularly in LC Stage I, which likely reflecting the low abundance of tumor-derived cfDNA into the circulation (142). Similarly, *Li Y and colleagues* developed the HIFI (High-performance Infrastructure For MultIomics) platform, based on low-Whole Genome Sequencing (WGS), for early-stage LC detection and postoperative MRD monitoring. By integrating multiple cfDNA-derived features with ML-based algorithms, this approach combines long-fragment DNA sequencing enriched for hyper-methylated regions with circulating single molecule amplification and resequencing technology (cSMART 2.0). The HIFI demonstrated promising diagnostic and MRD monitoring performance, achieving a validation AUC of 0.912, a specificity of 96.3% for early-stage LC detection, and a sensitivity of 73.7% to MRD, while simplifying analytical workflow for implementation in clinical practice (143).

Among multimodal approaches, fragmentomics has emerged as promising complementary strategy in detecting tumor-associated alterations in cfDNA profiles, such as fragment length distributions, nucleosomal patterns, and terminal/end-motif signatures revealing tumor-specific chromatin organization (144).

Furthermore, Korean researchers have developed a tumor-origined fragments (TOFs) approach integrating cfDNA methylation profiles with fragmentomics features for the detection of LC. This strategy combines read-level ctDNA quantification covering 6,243 CpG markers, identified by comparing tumor tissues with healthy blood and tissue samples, with cfDNA fragmentation patterns. In a cohort of 298 plasma samples (201 LC patients and 97 controls), the TOF model achieved an AUC of 0.98 and accurately classified both early-stage NSCLC and SCLC cases, demonstrating that the integration of fragmentomic and methylation insights may enhance tumor signal detection in ctDNA-based assays (145).

*Kim M* et al. found that a deep learning model that merges cfDNA methylation signatures and fragment size features can help to discriminate LC patients from healthy individuals. Using 366 selected methylation markers derived from The Cancer Genome Atlas/Gene Expression Omnibus (TCGA/GEO) datasets and next-generation EM-seq data, the prediction model yielded an AUC of 0.87 (81.5% accuracy) in a cohort including 142 LC samples and 56 control samples. Moreover, the model demonstrated the ability to detect low tumor fractions, identifying approximately 1% tumor fraction with 98% specificity and 0.1% tumor fraction with 80% specificity. These results highlight the potential value of combining methylation and fragment size features for improving the detection of low-tumor burden disease and enhancing biological informations obtained from cfDNA analysis (146).

Consistent with these findings, *Wang Y and colleagues* demonstrated that integrating epigenetic and fragmentomic features of cfDNA into a ML framework enables highly accurate non-invasive diagnosis of LC in a prospective-multicenter cohort of patients with suspicious lung nodules. By profiling cfDNA through chromatin immunoprecipitation sequencing, reduced-representing bisulfite sequencing, and low-coverage WGS resolution, the authors identified 609 epigenetically informed genes whose fragmentomic signatures were incorporated into a predictive model. This model showed an AUC of 0.94, with a sensitivity of 90.4%, and a specificity of just over 83% in the independent validation cohort. Notably, this approach showed promising performance in the early-stage disease, including a sensitivity above 95% for stage I LC and 96.2% for minimally invasive adenocarcinoma, thereby reinforcing the potential of integrated epigenetic-fragmentomic approaches for the earlier detection of LC in setting characterized by low tumor-derived cfDNA abundance (147).

The growing complexity of multi-omic datasets has driven the application of AI and ML approaches for multimodal data integration. The ASCEND-LUNG study (NCT04817046) evaluated a comprehensive LC based framework (PKU-LCSMS) combining cfDNA multi-omic analyses with AI-assisted imaging approaches for LC screening and management. In a two-stage prospective case-control cohort (n=465 for model development; n=291 for independent validation), the cfDNA methylation-based model achieved an AUC of 0.910 and outperformed models solely on protein and mutation biomarkers. The integration of methylation and protein features further improved performance, achieving an AUC of 0.963. In the independent cohort, the final screening model achieved a sensitivity of over 99%, although specificity remained limited (56%). For post-screening nodule management, an AI-assisted improved performance with CT imaging achieved a sensitivity of 81% and a specificity of 76%, underscoring the importance of adopting multi-omic and imaging approaches for early LC detection and pulmonary nodule stratification (148, 149).

Targeted methylation-based approach has been also used for the characterization of pulmonary nodule. In particular, PulmoSeek model, based on targeted DNA methylation sequencing, achieved an accuracy of 80.0% in distinguishing between benign and malignant nodules (150). Furthermore, the PulmoSeek Plus model, which integrates clinical/radiological features (CIBM model) with DNA methylation model (PulmoSeek), improved discrimination between benign and malignant lung nodules using two threshold values (0.65 and 0.89) and reduced unnecessary surgical procedures and treatment delays. In addition, the PulmoSeek Plus model demonstrated greater discriminatory power than the CIBM and PulmoSeek models, with an increase of 0.05 in the AUC (PulmoSeek Plus vs. CIBM, 95% CI 0.022–0.087, p=0.001; and PulmoSeek Plus vs. PulmoSeek, 0.018–0.083, p=0.002) (151),. Similarly, *Liang* et al. developed an integrative risk stratification model combining 40 cfDNA methylation biomarkers, age and five computed tomography (CT) imaging features. Specifically, the combined model significantly improved diagnostic performance, achieving an AUC of 0.90 (95% CI 0.86–0.94) in the internal cohort and to 0.89 (95% CI 0.85–0.92) in the external validation cohort, compared with 0.81 for the methylation-only model (95% CI 0.76–0.86) (113).

Taken together, integrated approaches combining cfDNA methylation, fragmentomics, additional molecular features, imaging parameters, and AI-based algorithms represent a promising strategy to enhance the scalability and clinical applicability of LB approaches in LC. Nevertheless, most ML-based models have been developed and validated in case-control cohorts enriched for disease prevalence, which may not fully reflect real-world screening populations. Prospective validation in large population-based cohorts and across diverse clinical settings will be essential before widespread clinical implementation.

### 6.2 Multimodal approach: a snapshot

Beyond early diagnosis, the incorporation of epigenetic LB into the detection of MRD following surgery and/or perioperative or adjuvant treatment represents a significant advance towards personalized disease monitoring. Conventional clinical and pathological parameters do not fully capture the heterogeneous risk of recurrence following curative treatment.

In this context, epigenetic LB has been shown to provide significant clinical value, even when compared with mutation-based ctDNA profiling. *Chen* et al. demonstrated that an approach based on tumor methylation status improved the identification of patients at risk of postoperative follow-up compared with strategies relying on somatic mutation detection. This advantage was particularly evident in subgroups with lower tumor burden, including stage I disease, and among patients with negative baseline ctDNA mutation status. In their study, patients were classified into different risk categories, according to cfDNA methylation profiles; higher timMRD scores were associated with increased risk of relapse and shorter disease-free survival (128). Therefore, when combined with radiological assessment and clinical parameters, cfDNA methylation profile may contribute to a more personalized follow-up strategy, allowing monitoring intensity and follow-up schedules to be tailored to individual recurrence risk.

Overall, multimodal approaches integrating epigenetic LB with clinical data and radiological features may represent a promising framework for shifting from population-based risk assessment toward more individualized strategies for both early detection and MRD-guided surveillance settings.

## 7 Critical appraisal of current evidence and unresolved challenges in cfDNA methylation-based LB: an overview

Although LB based on cfDNA methylation has shown promising results in a variety of clinical applications, interpreting the available evidence requires careful consideration of several methodological limitations. One of the main challenges is the considerable heterogeneity among published studies, which differ in cohort composition, distribution of disease stages, sample size, methylation profiling platforms, marker selection strategies, and bioinformatics pipelines. These differences limit direct comparisons between studies and may contribute to the variability in reported diagnostic performance (152, 153). It is important to note that many studies evaluating methylation signatures have been conducted on retrospective cohorts or using case-control designs, often including patients with already diagnosed cancer and a relatively high tumor burden. Therefore, the reported sensitivity and specificity values may not fully reflect performance in real-world clinical settings, particularly in early-stage LC, where tumor-derived cfDNA is extremely limited and biological variability is greater (63). Furthermore, several recently proposed methylation classifiers are based on complex machine learning algorithms developed using relatively small or highly selected cohorts, which raises concerns regarding model overfitting, reproducibility, and external validation on independent populations. Another unresolved issue concerns the optimal balance between discovery-oriented approaches and clinically applicable approaches. Genome-wide strategies provide a comprehensive characterization of the methylome and enable the identification of new tumor-associated signatures; however, their complexity may limit their applicability in routine clinical practice (152, 154). In contrast, targeted methylation panels offer greater analytical feasibility but may detect only a limited fraction of tumor-associated epigenetic alterations, potentially compromising their reliability across different histological and molecular subtypes (64, 155). It is worth noting that direct comparisons between different methylation platforms, conducted on standardized cohorts, remain scarce, making it difficult to determine whether the reported differences in performance reflect true analytical superiority or simply methodological variability. Furthermore, although methylation signatures provide valuable information on tumor identity and tissue of origin, their generalizability across different populations and tumor contexts remains an open question. Differences in tumor biology, epigenetic heterogeneity, and the background composition of cfDNA can influence test performance and highlight the need for cautious interpretation when transferring individual signatures across clinical settings.

Among these sources of biological variability, tobacco smoking deserves particular consideration because it induces widespread and partly persistent DNA methylation alterations in both blood and lung tissue, including changes involving *AHRR*, *MYO1G*, and *CYP1A1*. Some smoking-associated signatures remain detectable many years after cessation and may therefore reflect cumulative tobacco exposure rather than tumor-specific biology. Because a substantial proportion of circulating cfDNA originates from hematopoietic cells, these exposure-related signals may be detected independently of tumor-derived methylation, potentially affecting assay specificity and diagnostic accuracy when cases and controls differ in smoking status or intensity. Conversely, methylation-derived measures of smoking exposure may support lung cancer risk stratification but should not be interpreted as cancer-specific diagnostic signals. Therefore, cfDNA methylation studies should account for smoking status, pack-years, and time since cessation; include appropriately smoking-matched controls; and report stratified or adjusted performance across current, former, and never-smokers (156, 157).

Finally, although several studies have demonstrated encouraging diagnostic performance, evidence demonstrating the added clinical value of methylation-based tests compared with existing diagnostic strategies, including LDCT and mutation-based ctDNA testing (153, 158), remains limited and should be confirmed by prospective clinical trials with adequate statistical power.

## 8 Current challenges and concluding remarks

Epigenetic characterization of cfDNA is gaining more importance as powerful tool in LB, allowing for non-invasive analysis of tumor biology that moves way further than just genetic alterations (20). In the era of epigenomic modifications, DNA methylations are among the most extensively studied and clinically promising epigenetic biomarkers, as it provides biologically significant signals that reflect the progression of the tumor. The possibility of integrating cfDNA-based methodologies into a wider epigenetic framework, as well as bisulfite-based, bisulfite-free enrichment, and restriction enzyme-based approaches, covering not only DNA methylation but also fragmentomics and nucleosomics may further improve sensitivity and provide additional information on the origin of the tumor, prognosis, and response to treatment at different stages of SCLC, although these applications require further clinical validation (159).

However, its clinical translation is currently hindered by several biological, technical, and clinical challenges. A major limitation is the low fraction of ctDNA, particularly in early-stage disease and MRD settings, where tumor-derived signals may be diluted by a high background of non-tumor cfDNA, potentially reducing analytical sensitivity and increased the risk of false-negative results (160). This challenge is further influenced by inter- and intra-tumoral heterogeneity, generating variable methylation landscapes that may not be adequately captured by a single blood sample, particularly in LC where spatial and temporal heterogeneity is well documented. Moreover, cfDNA is derived from multiple tissue sources, including hematopoietic cells, introducing additional complexity in signal interpretation. Therefore, accurate tissue-of-origin prediction and computational deconvolution strategies are required. Importantly, methylation patterns are also affected by age, sex, inflammatory status, and environmental exposures, increasing confounding signals and the risk of false-positive (153, 161–163).

From a technical perspective, cfDNA fragmentation and low abundance remain major analytical challenges. Although bisulfite conversion is one of the most widely used strategies for methylation profiling, the chemical treatment can introduce DNA degradation and loss, limiting low-input performance. Alternative approaches, including enzymatic conversion and affinity-based enrichment methods, may offer potential advantages but present limitations related to genomic coverage, resolution, and reproducibility (76). Moreover, WGBS approach provides comprehensive methylome characterization but remains limited by high cost and computational requirements, whereas targeted panels are more feasible but may miss relevant and broader epigenetic alterations outside predefined regions. The lack of standardized protocols across cfDNA workflows and bioinformatics pipelines, further complicates cross-study comparison and affect reproducibility. Bioinformatic analysis remains a bottleneck, requiring sophisticated pipelines and integration with ML approaches. However, predictive models developed from small or heterogeneous datasets could be considered vulnerable to overfitting (15, 153, 164).

Clinically, methylation-based assays remain under prospective evaluation in early-stage LC, limiting their utility as standalone screening tools. False-positive methylation signals without clear tumor localization may lead to unnecessary diagnostic procedures, raising concerns about overdiagnosis and cost-effectiveness (165). Additional limitations could include the lack of availability of large, prospective validation studies and real-world evidence, as well as economic and infrastructural barriers linked to high cost of NGS-based platforms and technical requirements.

Future progress is expected to rely on the development of robust, integrative multi-omic models that combine DNA methylation with complementary molecular features, along with improved machine learning approaches and scalable analytical workflows. However, the clinical applicability of these strategies will depend on external validation, model interpretability, the prevention of overfitting, and the demonstration of reproducibility across independent cohorts (166). In this context, another emerging paradigm in translational oncology is the identification of clinically relevant signatures within the multi-dimensional data of LB, yet interpretability and external validation would remain critical for their real-world application (167).

Overall, LB using cfDNA methylation is shifting toward a more integrated, data-driven approach with significant potential to improve early diagnosis, molecular characterization, and monitoring of LC. However, despite encouraging results from retrospective studies and early clinical validation cohorts, current evidence is still insufficient to support routine clinical implementation (168, 169). Major unresolved challenges include the limited sensitivity in settings with extremely low tumor burden, biological heterogeneity, variability in methylation profiles, lack of standardized pre-analytical and analytical workflows, and the need for robust quality-control criteria (170, 171). Furthermore, large prospective multicenter studies are required to confirm clinical utility, define optimal applications across different disease stages, and demonstrate cost-effectiveness compared with current diagnostic strategies. Overcoming these biological, technical, and clinical barriers will be essential before cfDNA methylation assays can be incorporated into standard LC management.
